# Heuristic shortest hyperpaths in cell signaling hypergraphs

**DOI:** 10.1186/s13015-022-00217-9

**Published:** 2022-05-26

**Authors:** Spencer Krieger, John Kececioglu

**Affiliations:** Department of Computer Science, The University of Arizona, Tucson, Arizona 85721 USA

**Keywords:** Systems biology, cell signaling networks, reaction pathways, directed hypergraphs, shortest hyperpaths, efficient heuristics, hyperpath enumeration

## Abstract

**Background:**

Cell signaling pathways, which are a series of reactions that start at receptors and end at transcription factors, are basic to systems biology. Properly modeling the reactions in such pathways requires *directed hypergraphs*, where an edge is now directed between two sets of vertices. Inferring a pathway by the most parsimonious series of reactions corresponds to finding a *shortest hyperpath* in a directed hypergraph, which is NP-complete. The current state-of-the-art for shortest hyperpaths in cell signaling hypergraphs solves a mixed-integer linear program to find an optimal hyperpath that is restricted to be acyclic, and offers no efficiency guarantees.

**Results:**

We present, for the first time, a heuristic for general shortest hyperpaths that properly handles *cycles*, and is guaranteed to be *efficient*. We show the heuristic finds provably optimal hyperpaths for the class of singleton-tail hypergraphs, and also give a practical algorithm for tractably generating all source-sink hyperpaths. The accuracy of the heuristic is demonstrated through comprehensive experiments on all source-sink instances from the standard NCI-PID and Reactome pathway databases, which show it finds a hyperpath that *matches* the state-of-the-art mixed-integer linear program on over 99% of all instances that are acyclic. On instances where only cyclic hyperpaths exist, the heuristic *surpasses* the state-of-the-art, which finds no solution; on every such cyclic instance, enumerating all source-sink hyperpaths shows the solution found by the heuristic was in fact *optimal*.

**Conclusions:**

The new shortest hyperpath heuristic is both *fast* and *accurate*. This makes finding source-sink hyperpaths, which in general may contain cycles, now practical for real cell signaling networks.

**Availability:**

Source code for the hyperpath heuristic in a new tool we call Hhugin (as well as for hyperpath enumeration, and all dataset instances) is available free for non-commercial use at http://hhugin.cs.arizona.edu.

## Background

Cell signaling pathways are cornerstones of molecular and cellular biology. They underly cellular communication, govern environmental response, and their perturbation has been implicated in the cause of many diseases [1]. While signaling pathways have classically been modeled as ordinary graphs, using directed or undirected edges to link pairs of interacting molecules  [2, 3], both Klamt, Haus and Theis [4] and Ritz, Tegge, Kim, Poirel and Murali  [5] have shown that ordinary graphs cannot adequately represent cellular activity that involves the assembly and disassembly of protein complexes, or multiway reactions among such complexes.

Directed *hypergraphs* are generalizations of ordinary graphs where an edge, now called a *hyperedge*, is directed from one set of vertices, called its *tail*, to another set of vertices, called its *head*. Hypergraphs have been used to model many cellular processes  [4–12]. In particular, a biochemical reaction that involves multiple reactants, all of which must be present for the reaction to proceed, and that results in multiple products, all of which are produced upon its completion, is correctly captured by a single hyperedge directed from its set of reactants to its set of products. Despite hypergraphs affording more faithful models of reaction networks, the lack of practical hypergraph algorithms has hindered their potential for properly representing and reasoning about molecular reactions.

Biologically, a typical cell-signaling pathway consists of membrane-bound receptors that bind to extracellular ligands, triggering intracellular cascades of reactions, culminating in the activation of transcriptional regulators and factors  [13]. Computationally, treating receptors as sources, and transcription factors as targets, finding the most efficient way to synthesize a particular transcription factor from a set of receptors maps to the shortest hyperpath problem we consider here: Given a cell-signaling network whose reactants and reactions are modeled by the vertices and weighted hyperedges of a directed hypergraph, together with a set of sources and a target, find a hyperpath consisting of hyperedges from the sources to the target of minimum total weight. We briefly summarize prior work on related problems next.

### Related work

Hypergraphs have been studied in the algorithms community [14–16], and applied within systems biology to metabolic networks  [17–20] and cell-signaling networks  [12, 21–23].

In the field of algorithms, Italiano and Nanni [14] first proved that finding a shortest source-sink hyperpath is NP-complete, even when hyperedges have a single head vertex. In a seminal paper that is the source for much of the subsequent work on hypergraphs, Gallo, Longo, Pallottino and Nguyen [15] explore special cases of hypergraphs, and define several versions of hyperpaths, including what they call a *B*-path (though see the correction of Nielsen and Pretolani [24]), which is essentially equivalent to our definition of hyperpath (given in the following section on shortest hyperpaths in directed hypergraphs). They show the vertices reachable from a source vertex in a hypergraph can be found in time linear in the total size of the tail and head sets of all hyperedges, give an efficient algorithm for a variant of shortest hyperpaths with a so-called additive cost function, and prove that finding a minimum cut in a hypergraph is NP-complete. Ausiello and Laura [16] survey results on hypergraphs whose hyperedges have singleton head sets, and note that a consequence of the NP-completeness reduction [14] for shortest hyperpaths from the set cover problem is that, unless $\text {P} \!=\! \text {NP}$, no approximation algorithm can exist for shortest hyperpaths on hypergraphs of *n* vertices with approximation ratio $\bigl ( 1 \!-\! o(1) \bigr ) \ln n$.

In metabolic networks, Cottret, Milreu and Acuña et al. [17] examine the minimum precursor problem: given a hypergraph *G*, a set of sources *S*, and a set of targets *T*, find a source subset $P \subseteq S$ of minimum cardinality that has a factory from *P* to *T*, where a *factory* is a set of hyperedges that produce targets *T* from precursor set *P* while satisfying weaker ordering constraints on hyperedges than required by hyperpaths. They show this problem is NP-complete, and give an algorithm that enumerates all minimal precursor sets whose factory is acyclic. Acuña, Milreu and Cottret et al. [18] subsequently enumerate all minimal precursor sets allowing cycles. Andrade, Wannagat and Klein et al. [19] extend these algorithms to accommodate stoichiometry and conserve intermediate metabolites within the factory. Carbonell, Fichera, Pandit and Faulon [20] give an efficient algorithm to find a source-sink hyperpath if one exists—irrespective of its length—and prove that finding any hyperpath that must contain a specified set of hyperedges is NP-complete. They also offer an approach to hyperpath enumeration that relies on solutions to this NP-complete problem, for which they employ a heuristic.

In cell-signaling networks, Ritz, Avent and Murali  [12, 21] were the first to solve the shortest *acyclic* hyperpath problem by formulating it as a mixed-integer linear program (MILP)—the current state-of-the-art for shortest hyperpaths—and showed that in practice, optimal acyclic hyperpaths can be found even for large cell-signaling hypergraphs. Their formulation does not extend to hyperpaths with cycles, and requires exponential time in the worst-case (which may be unavoidable, as the acyclic problem remains NP-complete). Recently, Franzese, Groce, Murali and Ritz [22] defined a parameterized notion of connectivity that interpolates between hyperpath- and ordinary-path-connectivity, while Schwob, Zhan and Dempsey [23] modified the acyclic MILP of Ritz et al. [21] to include time-dependence among reactions.

### Our contributions

In contrast to prior work, we present a heuristic for shortest hyperpaths that handles cycles, is worst-case efficient, and finds hyperpaths that are demonstrably optimal or close to optimal in real cell-signaling hypergraphs. In more detail, we make the following contributions.

- We present an *efficient heuristic* for shortest hyperpaths, that on a hypergraph of size $\ell$, which measures the total cardinality of all hyperedge tail and head sets, with *m* hyperedges that are doubly-reachable from the source and sink vertices, and *k* defined analogously to $\ell$ over these doubly-reachable hyperedges, runs in $O(\ell \,+\, m^2 \, k)$  time.
- We prove that the heuristic finds an *optimal* shortest hyperpath for the class of *singleton-tail hypergraphs*, where the tails of all hyperedges in the hypergraph are single vertices.
- We also give a practical algorithm for *hyperpath enumeration* that generates all possible source-sink hyperpaths, allowing us to tractably measure how close our heuristic is to the optimum.
- Our heuristic *matches the state-of-the-art* MILP for shortest acyclic hyperpaths on over 99% of all instances from two standard databases of cell-signaling pathways.
- Our heuristic *surpasses the state-of-the-art* on instances where every source-sink hyperpath is cyclic, and hence the MILP finds no solution. On all such cyclic biological instances, our hyperpath enumeration algorithm verified that the heuristic was in fact *optimal*.

To our knowledge, this heuristic is the *first in the literature* for shortest source-sink hyperpaths in general directed hypergraphs, where hyperedges have arbitrary tail and head sets, and the length of a hyperpath is the sum of the weights of its hyperedges.

We note that the worst-case efficiency and subclass optimality of the heuristic highlighted in the first two points above show that the shortest hyperpaths problem is polynomial-time solvable for singleton-*tail* hypergraphs—in contrast to its NP-completeness for singleton-*head* hypergraphs  [14]—which does not appear to have been observed before in the literature  [16]. Furthermore, while prior work has developed specialized algorithms that are tailored to shortest hyperpaths under so-called *additive* cost functions [15]—which also handle singleton-tail hypergraphs—in distinction, we give a general heuristic for arbitrary hypergraphs under the *non-additive* cost function of total weight of the hyperpath, that as a consequence is optimal for the special case of singleton-tail hypergraphs.

Source code for an implementation of the shortest hyperpath heuristic in a new tool we call Hhugin [25] (short for “hypergraph heuristic for general shortest source-sink hyperpaths”), as well as the hyperpath enumeration algorithm and all dataset instances, is available free for non-commercial use at http://hhugin.cs.arizona.edu.

### Plan of the paper

The next section defines the general shortest hyperpath problem, allowing cycles. The following section then presents our heuristic for shortest hyperpaths, analyzes its time complexity, shows it returns a feasible solution whenever one exists, and proves it finds optimal solutions for singleton-tail hypergraphs. The next section gives our algorithm for generating all source-sink hyperpaths, proves its correctness, and analyzes its time complexity. The subsequent section compares the heuristic, through experiments on all source-sink instances from standard databases, to the state-of-the-art MILP for acyclic instances, or to the optimum of all enumerated hyperpaths for cyclic instances, and discusses three examples of cyclic shortest hyperpaths in cell signaling networks. Finally, the last section concludes, and provides directions for further research.

## Shortest hyperpaths in directed hypergraphs

**Fig. 1:**
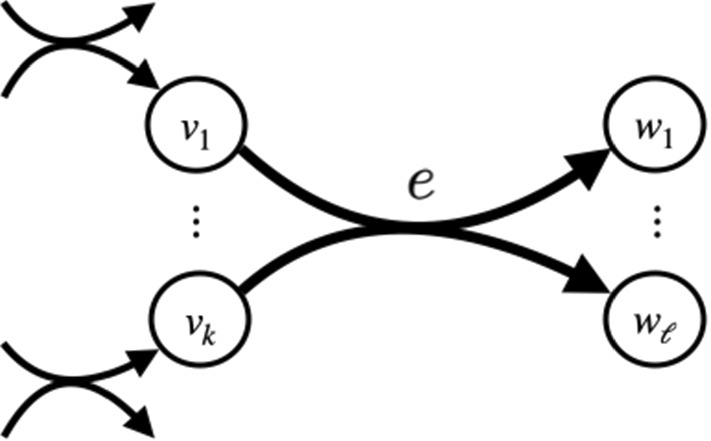
Hyperedge. A hyperedge *e* with $\mathrm {tail}(e) = \{v_1, \ldots , v_k\}$ and $\mathrm {head}(e) = \{w_1, \ldots , w_\ell \}$. To use *e* in a hyperpath *P*, every vertex $v_i \in \mathrm {tail}(e)$ must have a preceding hyperedge *f* in *P* with $v_i \in \mathrm {head}(f)$

A directed hypergraph is a generalization of an ordinary directed graph, where an edge, instead of touching two vertices, now connects two subsets of vertices. Formally, a directed *hypergraph* is a pair (*V*, *E*), where *V* is a set of vertices, and *E* is a set of directed *hyperedges*. (The literature sometimes uses the term *hyperarc* for an edge in a directed hypergraph, but we prefer the simpler term *hyperedge*—just as the term edge is conventionally used for both directed and undirected ordinary graphs. We will occasionally abbreviate the term hyperedge to simply *edge*, when it is clear that the context is with respect to a directed hypergraph.) Each hyperedge $e \in E$ is an ordered pair (*X*, *Y*), where both $X,Y \subseteq V$ are vertex subsets. Edge *e* is directed from set *X* to set *Y*. We call set *X* the *tail* of *e*, and set *Y* the *head* of *e*, and refer to these sets by the functions $\mathrm {tail}(e) = X$ and $\mathrm {head}(e) = Y$. We also refer to the *in-edges* of vertex *v* by $\text {in}(v) := \{ e \!\in \! E \,:\, v \in \mathrm {head}(e) \}$, and the *out-edges* of *v* by $\text {out}(v) := \{ e \!\in \! E \,:\, v \in \mathrm {tail}(e) \}$. Figure 1 shows a directed hyperedge.

In ordinary directed graphs, a path from a vertex *s* to a vertex *t* is a sequence of edges starting from *s* and ending at *t*, where for consecutive edges *e* and *f* in the sequence, the preceding edge *e* must enter the vertex that the following edge *f* leaves. We say *t* is reachable from *s* when there is such a path from *s* to *t*.

In generalizing these notions to directed hypergraphs, the conditions both for when a hyperedge can follow another in a hyperpath, and when a vertex is reachable from another, become more involved. A hyperpath is again a sequence of hyperedges, but now for hyperedge *f* in a hyperpath, for every vertex $v \in \mathrm {tail}(f)$, there must be some hyperedge *e* that precedes *f* in the hyperpath for which $v \in \mathrm {head}(e)$. Reachability is captured by the following notion of superpath.

### Definition 1

**(Superpath)** In a directed hypergraph (*V*, *E*), an *s*,*t*-*superpath*, for vertices $s,t \in V$, is an edge subset $F \subseteq E$ such that the hyperedges of *F* can be ordered $e_1, e_2, \ldots , e_k$, where

(i) $$\mathrm {tail}(e_1) \,=\, \{s\}$$,
(ii) for each  $1 < i \le k$,
  $$\begin{aligned} \mathrm {tail}(e_i) \;\;\subseteq \;\; \{s\} \;\cup \; \bigcup _{1 \le j < i} \mathrm {head}(e_j) \, , \end{aligned}$$
(iii) and  $$t \,\in \, \mathrm {head}(e_k)$$.

For an *s*,*t*-superpath, we call *s* its *source*, *t* its *sink*, and we say *t* is *reachable* from *s*.${\square}$

We can now define hyperpaths in terms of superpaths. Recall that a set *S* is *minimal* with respect to some property *X* if *S* satisfies *X*, but no proper subset of *S* satisfies *X*.

### Definition 2

**(Hyperpath)** An  *s*,*t*-*hyperpath* is a minimal *s*,*t*-superpath.${\square}$

In other words, a hyperpath *P* is a superpath for which removing any edge $e \in P$ leaves a subset $P - \{e\}$ that is no longer a superpath. Essentially, hyperpaths eliminate unnecessary edges from superpaths. Figures 7, 8, and 9 later show examples of hyperpaths.

We say a hyperpath *P* contains a *cycle* if, for every ordering $e_1, \ldots , e_k$ of its hyperedges satisfying properties (i)–(iii) in the definition of superpath, *P* contains some hyperedge *f* with a vertex in $\mathrm {head}(f)$ that also occurs in $\mathrm {tail}(e)$ for an earlier hyperedge *e* in the ordering. While in ordinary graphs a minimal *s*,*t*-path can never contain a cycle, in hypergraphs an *s*,*t*-hyperpath can in fact contain cycles, as shown in our later section on biological examples.

We can now define the shortest hyperpath problem. For an edge weight function $\omega (e)$, we extend $\omega$ to edge subsets $F \subseteq E$ by $\omega (F) \,:=\, \sum _{e \in F} \, \omega (e)$.

### Definition 3

**(Shortest Hyperpaths)** The *Shortest Hyperpaths* problem is the following. Given a directed hypergraph (*V*, *E*), a positive edge weight function $\omega : E \!\rightarrow \! \mathcal {R}^+$, source $s \in V$ and sink $t \in V$, find an *s*,*t*-hyperpath $P \subseteq E$ of minimum total weight  $\omega (P)$.${\square}$

Note that for positive edge weights, Shortest Hyperpaths is equivalent to finding an *s*,*t*-*superpath* of minimum total weight.

Shortest Hyperpaths with a single source and sink vertex also captures more general versions of the problem with *multiple sources* and *multiple sinks*, as follows. To find a hyperpath that starts from a set of sources $S \subseteq V$, simply add a new source vertex *s* to the hypergraph together with a single hyperedge $(\{s\},S)$ of zero weight, and equivalently find a hyperpath from the single source *s*. To find a hyperpath that reaches *all* vertices in a set of sinks $T \subseteq V$, add a new sink vertex *t*, a zero-weight hyperedge $(T,\{t\})$, and equivalently find a hyperpath to the single sink *t*. To find a hyperpath that reaches *some* vertex in a set of sinks $T \subseteq V$, add new sink vertex *t*, zero-weight hyperedges $(\{v\},\{t\})$ from all $v \in T$, and again equivalently find a hyperpath to the single sink *t*. Thus versions of shortest hyperpaths with multiple sources and sinks can be reduced to the problem with a single source and sink.

Shortest Hyperpaths is NP-complete [14] (even for acyclic hypergraphs with singleton head sets), so we likely cannot efficiently compute shortest hyperpaths in the worst-case. The next section presents an efficient heuristic for shortest hyperpaths that is highly accurate at finding demonstrably optimal or near-optimal hyperpaths in real cell-signaling hypergraphs.

## An efficient shortest hyperpath heuristic

We now give a fast heuristic for Shortest Hyperpaths that always finds an *s*,*t*-hyperpath if one exists. While the heuristic is not guaranteed to find a shortest *s*,*t*-hyperpath in general, our later experiments on real cell-signaling hypergraphs show it quickly finds a hyperpath that is optimal or remarkably close to optimal on the vast majority of instances in comprehensive experiments over the two standard cell-signaling databases in the literature. Furthermore, we will prove that the heuristic is guaranteed to find a shortest *s*,*t*-hyperpath for the class of singleton-tail hypergraphs, where the tail-sets of all hyperedges are single vertices.

We present the heuristic by providing detailed *pseudocode* at a level that can be directly implemented, as the heuristic is carefully designed and many of its component algorithms are surprisingly tricky to implement correctly. After describing the heuristic, we give a *time analysis* that shows it is always efficient, prove its *feasibility*, and then show that it finds *optimal* hyperpaths for singleton-tail hypergraphs.

While at a high level the heuristic has some aspects in common with Dijkstra’s algorithm for single-source shortest paths in an ordinary directed graph (see [26, pp. 658–659])—in that the heuristic maintains a heap of elements prioritized by estimated path lengths—it has significant differences. In contrast to Dijkstra’s algorithm, the heuristic is edge-based, rather than vertex-based, and the heap maintains hyperedges *e* prioritized by the length of the shortest known hyperpath from the source *s* to edge *e*, which will be formally defined later. Also in contrast to Dijkstra’s algorithm, maintaining a single in-edge to a vertex no longer suffices for recovering a path back to source *s*; instead, recovering an *s*,*t*-hyperpath now requires the heuristic to maintain a set of in-edges to each hyperedge *e* that are candidates for the final edges on the path from *s* to *e*. Furthermore, the total length of a hyperpath *P* to *e* is no longer a simple function (like a minimum or a sum) of the lengths of hyperpaths to the in-edges of *e* in *P* that cover the tail of *e*, since the constituent hyperpaths within *P* to these in-edges of *e* can have arbitrarily-complicated sharing of hyperedges. Simply determining the length of the best recovered hyperpath for a hyperedge *e* on the heap, using these stored in-edges to each hyperedge, is itself now a hard combinatorial problem, which the heuristic tackles by a carefully-constructed greedy procedure.

The overall structure of the heuristic is a breadth-first search over the hyperedges *e* reachable from source *s*, ordered by the estimated length of the shortest hyperpath from *s* to *e*. (Admittedly a shortest *s*,*t*-hyperpath *P* is not necessarily composed of shortest hyperpaths from *s* to individual hyperedges *e* in *P*, which is partly why this approach is a heuristic.) The search repeatedly invokes a greedy procedure to recover the currently best-known hyperpath to *e* in order to evaluate its length. As hyperpaths are by definition minimal superpaths, to determine minimality this greedy recovery procedure repeatedly tests reachability of hyperedges. Moreover, for efficiency, the overall breadth-first search proceeds over a smaller subgraph of the input hypergraph that only contains hyperedges that are reachable both from source *s* and in reverse from sink *t*. Hence at base, the heuristic builds upon fast algorithms for computing reachability in a hypergraph.

Accordingly, to present the heuristic, we first give pseudocode for these fundamental algorithms for directed reachability. These algorithms use the following terminology of *forward-reachable*, *backward-traceable*, and *doubly-reachable*, which we define next.

### Definition 4

**(Reachability and Traceability)** Vertex *v* is *forward reachable* from source *s* in hypergraph *G* if there is an *s*,*v*-superpath in *G*. Hyperedge *e* is *forward reachable* from *s* if all vertices $v \in \mathrm {tail}(e)$ are forward reachable from *s*.

Vertex *v* is *backward traceable* from sink *t* if $v = t$, or recursively if $v \in \mathrm {tail}(e)$ for an edge *e* where some ${w \in \mathrm {head}(e)}$ is backward traceable from *t*. Hyperedge *e* is *backward traceable* from *t* if some ${v \in \mathrm {head}(e)}$ is backward traceable from *t*.

A vertex *v* or hyperedge *e* is *doubly reachable* if *v* or *e*, respectively, is both forward reachable from *s* and backward traceable from *t*.${\square}$

To describe the heuristic, it will also be convenient to extend the definitions of *superpath* and *hyperpath* to a path from a source *s* to a hyperedge *e*.

### Definition 5

**(Superpath and Hyperpath from Source to Hyperedge)** An *s*,*e*-*superpath* is an edge subset *S*  with $e \in S$ where all vertices in $\mathrm {tail}(e)$ are forward reachable from source *s* using hyperedges in *S*. An *s*,*e*-*hyperpath* is a minimal *s*,*e*-superpath.${\square}$

**Fig. 2:**
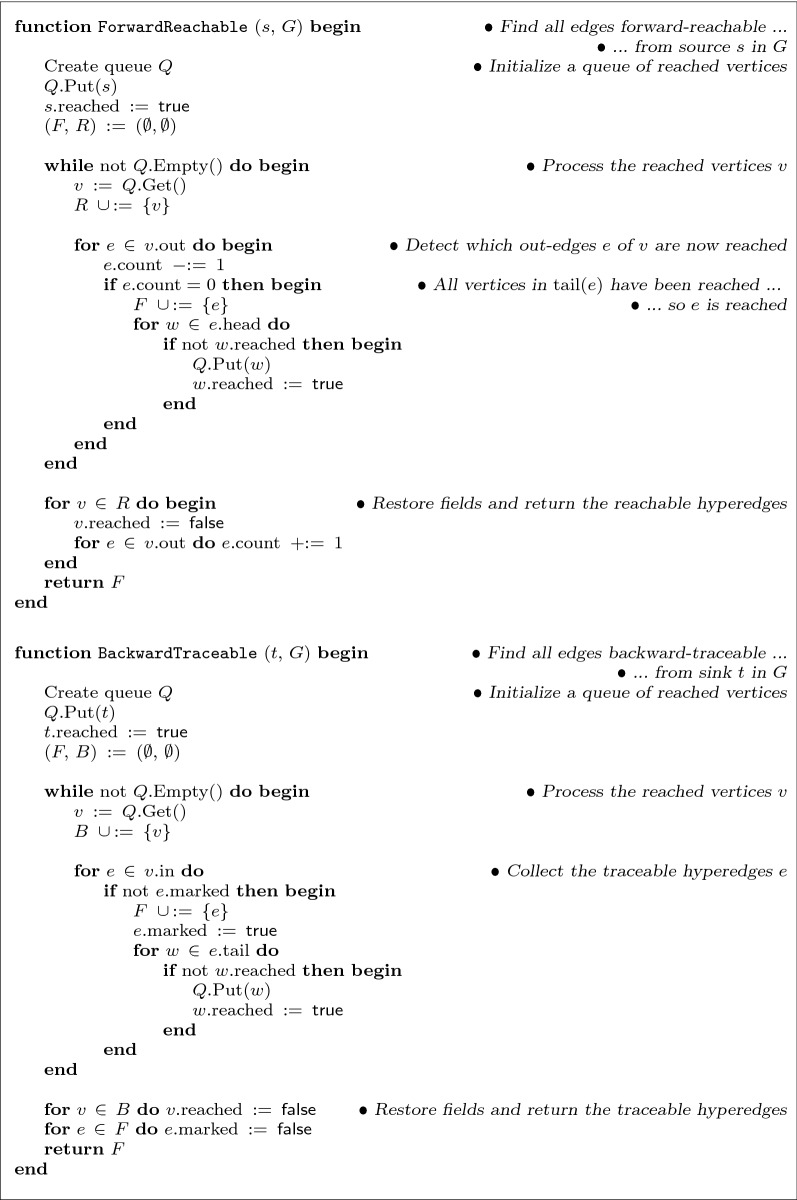
Reachability computations. Function ForwardReachable, given source vertex *s* in hypergraph *G*, returns all hyperedges *e* for which $\mathrm {tail}(e)$ is reachable by a hyperpath from *s*. Function BackwardTraceable, given sink vertex *t* in *G*, returns all hyperedges *e* for which some vertex $v \in \mathrm {head}(e)$ is backward-traceable from *t*. These functions assume fields *v*.reached, *e*.marked, and *e*.count have been initialized to false, false, and $|\mathrm {tail}(e)|$, respectively, for all *v* and *e* in *G*

**Fig. 3:**
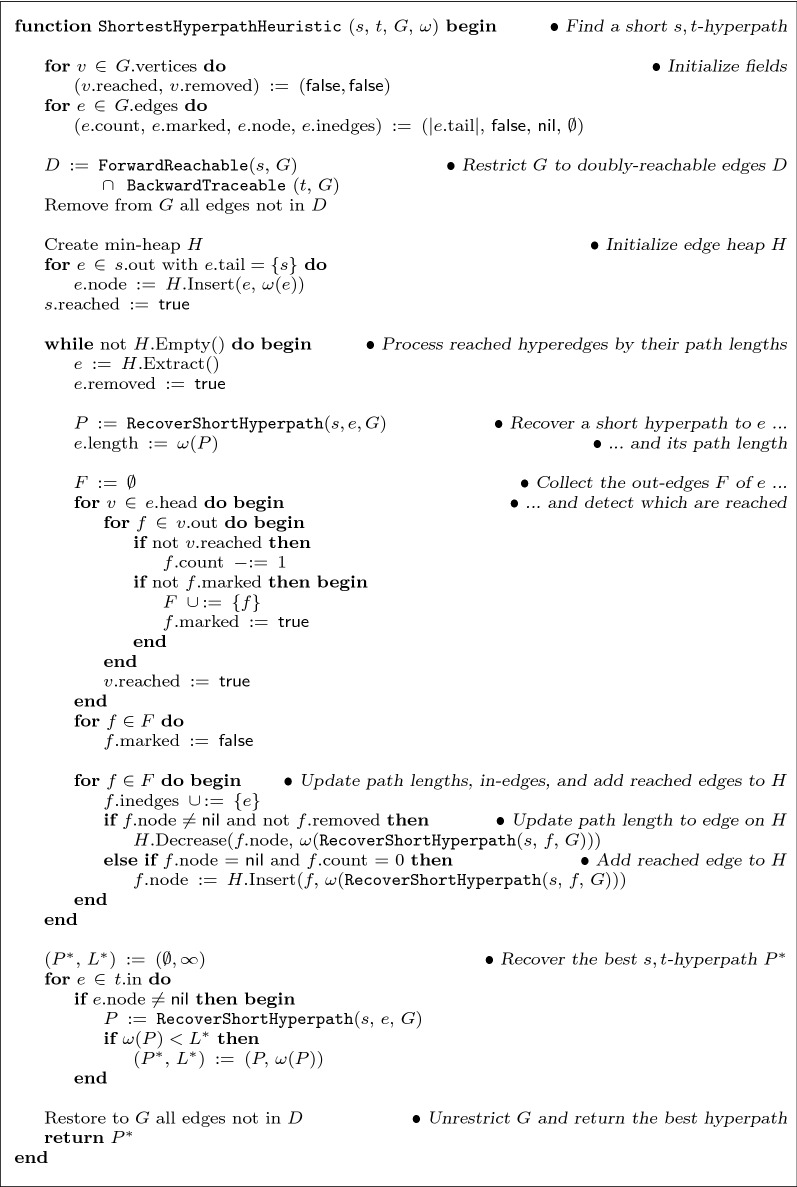
Efficient heuristic for shortest source-sink hyperpaths. Given source *s*, sink *t*, and edge weights $\omega$, function ShortestHyperpathHeuristic finds an *s*,*t*-hyperpath in hypergraph *G*, attempting to minimize its length. If no *s*,*t*-hyperpath exists, the empty path is returned. For doubly-reachable hyperedges *e*, the heuristic maintains fields *e*.length (the total weight of the shortest hyperpath found to *e*), and *e*.inedges (the subset of edges *f* with $\mathrm {head}(f)$ touching $\mathrm {tail}(e)$ where *f*.length is known), which are used in RecoverShortHyperpath to recover a short hyperpath to *e*

The pseudocode that we present accesses a hypergraph *G* through the fields *G*.vertices and *G*.edges. We access the tail-set and head-set of a hyperedge *e* through the fields *e*.head and *e*.tail. We access the set of in-edges and out-edges of a vertex *v* through the fields *v*.in and *v*.out. For a list *Q* that is handled as a queue, the operation *Q*.Put(*x*) appends item *x* to the rear of *Q*, while the operation *Q*.Get() removes and returns the item at the front of *Q*. For a min-heap *H*, the operation *H*.Insert(*x*, *k*) inserts item *x* with key *k* into *H*, and returns a reference *p* to the heap node containing this pair (*x*, *k*) in *H*; the operation *H*.Extract() removes and returns the item in *H* with minimum key; and the operation *H*.Decrease(*p*, *k*) takes a reference *p* to a heap node in *H* and decreases its key to *k* if *k* is smaller than its current key. All functions assume hypergraph *G* is passed by reference.

Figure 2 gives pseudocode for the two functions ForwardReachable and BackwardTraceable. Function ForwardReachable returns the set of all hyperedges that are forward reachable from source *s*, while function BackwardTraceable returns the set of hyperedges that are backward traceable from sink *t*. Function ForwardReachable uses the Boolean vertex field *v*.reached, and the integer edge field *e*.count, which it assumes have already been initialized to the values $v.\text {reached} = \textsf {false}$ for all $v \in V$ and $e.\text {count} = \bigl |\mathrm {tail}(e)\bigr |$ for all $e \in E$. Function BackwardTraceable also uses the Boolean edge field *e*.marked, which it similarly assumes is initialized to false for all *e*. (This initialization will be done once for hypergraph *G* in the shortest hyperpath heuristic, which allows these functions when called repeatedly to run in time bounded by just the size of the forward-reachable or backward-traceable subgraphs.) Function ForwardReachable uses the field *e*.count to detect when all vertices in $\mathrm {tail}(e)$ have been reached from *s*, and hence *e* is now reached from *s*. Function BackwardTraceable performs a similar but simpler computation in reverse from sink *t*. The worst-case time for both these functions is linear in the size of the subgraph they explore, as analyzed in the following section on the time-complexity of the heuristic.

**Fig. 4:**
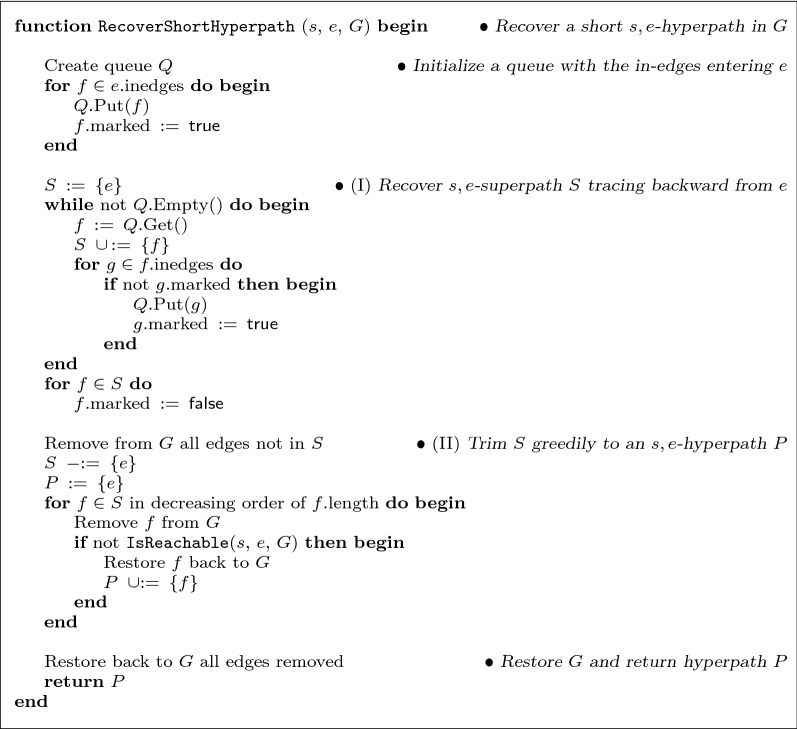
Recovering a short hyperpath from the source to a hyperedge. Given source vertex *s* and hyperedge *e*, function RecoverShortHyperpath returns an *s*,*e*-hyperpath *P* in hypergraph *G*, attempting to minimize its length. The edges of hyperpath *P* are greedily selected from an *s*,*e*-superpath *S* that is guaranteed to exist in *G*, where *S* is recovered by tracing backward from *e*

Figure 3 gives pseudocode for the function ShortestHyperpathHeuristic, our heuristic. Like Dijkstra’s shortest path algorithm for ordinary graphs, this function maintains a heap *H*, but in contrast to Dijkstra’s algorithm this is now a heap of hyperedges *e* (rather than a heap of vertices), which are prioritized by keys that are the best known estimate of the length of a shortest *s*,*e*-hyperpath. We refer to this estimate as the current *path length* for *e*. The heuristic starts from the out-edges of source *s*, and in a reaching computation repeatedly extracts from heap *H* the hyperedge *e* with minimum key. When hyperedge *e* is removed from *H*, the estimated path length for *e* is evaluated, and stored in field *e*.length. To compute this length estimate, it must construct the best *s*,*e*-hyperpath it can find, and evaluate its total weight. Of course, computing an optimal *s*,*e*-hyperpath is NP-complete, so it uses a greedy heuristic to construct this path by the function RecoverShortPath. This greedy path-construction heuristic consists of two steps: (1) recovering an *s*,*e*-superpath by recursively backward-traversing hyperedges that enter $\mathrm {tail}(e)$, followed by (2) finding a minimal subset of this superpath that is an *s*,*e*-hyperpath while attempting to minimize its total weight.

Figure 4 gives pseudocode for the function RecoverShortHyperpath that implements this greedy path-construction heuristic. For the first step, recovering the *s*,*e*-superpath *S* is done by recursively backward-traversing what we call *in-edges*: those hyperedges whose head-sets intersect the tail-set of a given hyperedge. Function ShortestHyperpathHeuristic maintains for a hyperedge *e* the field *e*.inedges, which stores the subset of in-edges *f* to *e* whose field *f*.length has been determined.

For the second step, function RecoverShortHyperpath attempts to find the minimum weight subset of *S* that is still a superpath by greedily considering hyperedges $f \in S$ for removal in decreasing order of *f*.length, which is the estimated total length of a shortest *s*,*f*-hyperpath. (Note this is more sophisticated than a naive greedy approach that simply removes hyperedges *f* in decreasing order of their edge-weight $\omega (f)$, which would degenerate to removing edges in random order in real cell-signaling networks where hyperedges typically all have unit weight, and hence would all be tied for removal.) This greedy process for trimming superpath *S* repeatedly tests whether $\mathrm {tail}(e)$ is still reachable from *s* on removing *f* by calling Boolean function IsReachable. Pseudocode for IsReachable is not given, but it simply implements a version of function ForwardReachable that halts and returns true as soon as it adds *e* to the set of hyperedges reachable from *s*, or returns false after collecting the entire reachable set without encountering *e*.

We note that most of the computation of the shortest hyperpath heuristic proceeds over a much smaller subgraph of the input hypergraph *G*: namely the subgraph induced by the hyperedges ${D \subseteq E}$ that are *doubly reachable* (both forward reachable from *s* and backward traceable from *t*). This preserves correctness, since hyperedges that are not doubly reachable cannot be on an *s*,*t*-hyperpath and can safely be ignored (as argued in the later section on feasibility of the heuristic in the proof of Theorem 2).

To summarize, the shortest hyperpath heuristic proceeds greedily like Dijkstra’s algorithm, but with some important differences: it maintains a heap of hyperedges prioritized by estimated shortest path lengths to tail-sets, records a set of potential in-edges to a given hyperedge used for recovering a hyperpath to the hyperedge, and recovering such a hyperpath now involves another greedy heuristic to find a minimal superpath of small total weight.

Our later section on experimental results shows this heuristic is remarkably *close to optimal* on real cell-signaling hypergraphs. Given that no practical exact algorithm exists for general shortest hyperpaths, we determine the optimum by enumerating all *s*,*t*-hyperpaths and taking the minimum of their lengths, using an algorithm we develop in the later section on tractably generating all source-sink hyperpaths.

We note for this heuristic that the inapproximability of the shortest hyperpath problem [16], together with the polynomial time analysis of the next subsection, imply that unless $\text {P} = \text {NP}$, the heuristic cannot be a constant-factor approximation algorithm for shortest hyperpaths.

In the following subsections, we first analyze the *running time* of the heuristic; then show it always finds a *feasible solution* whenever one exists; and finally prove it actually finds an *optimal solution* for the class of singleton-tail hypergraphs.

### Time complexity of the heuristic

We now bound the time complexity of the shortest hyperpath heuristic. Our analysis is in terms of the following parameters measured on a hypergraph, or an induced subgraph. For a hypergraph *G* with vertices *V* and hyperedges *E*, we denote its number of vertices and hyperedges by

$$\begin{aligned} n\,\,:=\,\, & {} \,|V| \, , \\ m\,\,:=\,\, & {} \,|E| \, . \end{aligned}$$

We also use the *size* parameter

$$\begin{aligned} \ell \;\;:=\;\; \sum _{e \,\in \, E} \Bigl ( \bigl |\mathrm {tail}(e)\bigr | \;+\; \bigl |\mathrm {head}(e)\bigr | \Bigr ) \, , \end{aligned}$$

and *degree* parameter

$$\begin{aligned} d \;\;:=\;\; \max _{v \,\in \, V} \, \Bigl \{\, \bigl |\text {in}(v)\bigr |, \, \bigl |\text {out}(v)\bigr | \,\Bigr \} \, . \end{aligned}$$

Note that in general, the space required to represent all hyperedges is $\Theta (\ell )$. We assume all tail and head sets are nonempty, and every vertex is touched by a hyperedge, which implies $m + n = O(\ell )$. When we need to refer to these measures for a particular hypergraph *G*, such as on an induced subgraph, we explicitly subscript the parameters by the specific hypergraph, such as $n_G, \ldots , d_G$, where these parameters are then measured in terms of the vertices and edges of the subscripted hypergraph *G*.

The running time of the shortest hyperpath heuristic may be expressed as a function of parameters measured on both the input hypergraph and its doubly-reachable subgraph (induced by the hyperedges that are simultaneously forward reachable from the source and backward traceable from the sink).

#### Theorem 1

**(Time complexity of the heuristic)** *The time complexity of the shortest hyperpath heuristic, in terms of the number of hyperedges* *m* *and size parameter* $\ell$ *for both the input hypergraph* *G* *and its doubly-reachable subgraph* *H*, *is*

$$\begin{aligned} O\Bigl ( \ell _G \,\,+\,\, \ell _H \, m_H^2 \Bigr ) \, . \end{aligned}$$

#### Proof

To bound the running time of the function ShortestHyperpathHeuristic, we analyze in turn its component functions  ForwardReachable, BackwardTraceable, and RecoverShortHyperpath. The running time of the reachability computations ForwardReachable and BackwardTraceable (in Fig. 2) can be expressed in an output-sensitive way in terms of the size of the edge sets they return.

For ForwardReachable, let $R \subseteq V$ be the set of vertices reachable from source *s*, and $F \subseteq E$ be the set of hyperedges reachable from *s* that are returned. The total time for ForwardReachable is dominated by the time for its main while-loop, which takes time $\Theta \bigl ( \, \sum _{v \in R} \, \bigl |\text {out}(v)\bigr | \,\,+\,\, \sum _{e \in F} \, \bigl |\mathrm {head}(e)\bigr | \, \bigr )$, or equivalently,

$$\begin{aligned} \Theta \biggl ( \, \sum _{e \,\in \, E} \, \bigl |\mathrm {tail}(e) \,\cap \, R\bigr | \,\,\,+\,\,\, \sum _{f \,\in \, F} \, \bigl |\mathrm {head}(f)\bigr | \, \biggr ) \,\,\,=\,\,\, O\bigl ( \ell _G \bigr ) \, . \end{aligned}$$

For BackwardTraceable, let $B \subseteq V$ be the set of vertices it reaches from sink *t*, and $F \subseteq E$ be the set of hyperedges traceable from *t* that are returned. A similar analysis shows the time for BackwardTraceable is

$$\begin{aligned} \Theta \biggl ( \, \sum _{e \,\in \, E} \, \bigl |\mathrm {head}(e) \,\cap \, B\bigr | \,\,\,+\,\,\, \sum _{f \,\in \, F} \, \bigl |\mathrm {tail}(f)\bigr | \, \biggr ) \,\,\,=\,\,\, O\bigl ( \ell _G \bigr ) \, . \end{aligned}$$

So the time for both ForwardReachable and BackwardTraceable on the input hypergraph *G* is $O\bigl (\ell _G\bigr )$— but can be bounded more tightly in terms of the subgraph of *G* they actually explore.

For the function RecoverShortHyperpath (in Fig. 4), when it is called by ShortestHyperpathHeuristic, all its computations are performed on *G* restricted to the edge subset $D \subseteq E$ of doubly-reachable hyperedges. We denote by hypergraph *H* the doubly-reachable subgraph induced by *D*.

In RecoverShortHyperpath, the time to recover *s*,*e*-superpath *S* by tracing back from *e* is at most

$$\begin{aligned} O\biggl ( \, \sum _{f \,\in \, S} \,\, \sum _{v \,\in \, \mathrm {tail}(f)} \,\, \bigl | \text {in}(v) \bigr | \, \biggr ) \,\,\,=\,\,\, O\Bigl ( d_H \, \ell _H \Bigr ) \, . \end{aligned}$$

The time to greedily trim superpath *S* to *s*,*e*-hyperpath $P \subseteq S$, in terms of cardinality $k = |S|$, is at most

$$\begin{aligned} O\Bigl ( m_H \,\,+\,\, k \,\log \, k \,\,+\,\, k \, \ell _H \Bigr ) \,\,\,=\,\,\, O\Bigl ( k \, \ell _H \Bigr ) \, . \end{aligned}$$

Thus the total time for RecoverShortHyperpath is

$$\begin{aligned} O\Bigl ( d_H \, \ell _H \Bigr ) \,\,+\,\, O\Bigl ( k \, \ell _H \Bigr ) \,\,\,=\,\,\, O\Bigl ( \ell _H \, m_H \Bigr ) \, . \end{aligned}$$

For the function ShortestHyperpathHeuristic (in Fig. 3), we break its time down into the following components. The time for the initialization, collecting the doubly-reachable edges *D* by calling ForwardReachable and BackwardTraceable, and restricting *G* to its subgraph *H* induced by *D*, is $O\bigl (\ell _G\bigr )$. The main while-loop executes for $m_H$ iterations, and spends $O\bigl (m_H \, \log \, m_H\bigr )$ time for all Extracts. The total time across all iterations to compute *s*,*e*-hyperpath *P* for all extracted edges *e* by calling RecoverShortHyperpath is $O\bigl (\ell _H \, m_H^2\bigr )$. The total time to collect the out-edges *F* for the extracted *e* across all iterations is $O\bigl ( \sum _{e \in D} \, \sum _{v \in \mathrm {head}(e)} \, \bigl | \text {out}(v) \bigr | \bigr ) \,=\, O\bigl (d_H \, \ell _H\bigr )$. The total time across all iterations for Decrease and Insert, which take *O*(1) amortized time per edge in *F* using a Fibonacci heap  (see [26, pp. 510–522]), is also $O\bigl (d_H \, \ell _H\bigr )$. The time to recover the best *s*,*t*-hyperpath $P^*$ is $O\bigl (d_H \, \ell _H \, m_H \bigr )$.

Finally, adding up the bounds for the above components, the total time for the shortest hyperpath heuristic is

$$\begin{aligned} O\bigl (\ell _G\bigr ) \,\,+\,\, O\bigl (m_H \,\log \, m_H\bigr ) \,\,+\,\, O\bigl (\ell _H \, m_H^2\bigr ) \,\,+\,\, O\bigl (d_H \, \ell _H\bigr ) \,\,+\,\, O\bigl (d_H \, \ell _H \, m_H\bigr ) \, , \end{aligned}$$

which is in turn $O\bigl (\ell _G \,+\, \ell _H \, m_H^2\bigr )$.${\square}$

Notice that the overall running time of the heuristic is dominated by the total time to recover short hyperpaths, which requires invoking RecoverShortHyperpath whenever the path length to a hyperedge is updated. This is necessary in hypergraphs, since in contrast to ordinary graphs the length of the hyperpath to a hyperedge can no longer be expressed as a simple function (such as a minimum or a sum) of the lengths of the hyperpaths to its in-edges.

As demonstrated in our later section on experimental results, for real biological instances the size of the doubly-reachable subgraph *H* is significantly smaller than the full input hypergraph *G*, so designing the heuristic to compute mainly over the much smaller hypergraph *H* yields a significant performance speedup in practice.

Next we show the heuristic always finds a feasible solution.

### Feasibility of the heuristic

The most basic property that a heuristic for a combinatorial optimization problem should satisfy is *feasibility*: that it always returns a feasible solution whenever one exists. In the context of Shortest Hyperpaths, a feasible solution is any *s*,*t*-hyperpath, while an optimal solution is a feasible solution of minimum total edge-weight.

For the hyperpath heuristic, we now show feasibility.

#### Theorem 2

**(Feasibility of the heuristic)**  *The shortest hyperpath heuristic finds a source-sink hyperpath whenever one exists. *

#### Proof

Function ShortestHyperpathHeuristic (in Fig. 3) first restricts the input hypergraph *G* to its doubly-reachable subgraph, consisting of the hyperedges *D* that are both forward reachable from source *s* and backward traceable from sink *t*. Note that functions ForwardReachable and BackwardTraceable (in Fig. 2) together correctly collect these doubly-reachable hyperedges *D*: function ForwardReachable explores breadth-first the hyperedges that are forward reachable from *s*, maintaining a counter for each hyperedge *e* that records the number of vertices in its tail that have not yet been reached from *s*, and detecting when *e* is reached by this counter hitting zero; while function BackwardTraceable directly implements Definition 4 of backward traceability from *t*.

Furthermore, we claim that when restricting to the doubly-reachable subgraph ${\widetilde{G}}$, the heuristic does not lose any hyperedges on source-sink hyperpaths. Note that any hyperedge *e* on an *s*,*t*-hyperpath *P* in the input hypergraph *G* is forward reachable from *s*: consider the ordering of hyperedges in *P* from Definition 1, and take the prefix of this ordering up through *e*; this prefix is an *s*,*e*-superpath, so *e* is by definition forward reachable from *s*. Note also that any *e* on *P* in *G* is backward traceable from *t* as well: if ${t \in \mathrm {head}(e)}$, backward traceability immediately holds; otherwise, in the ordering of *P* there must be a hyperedge *f* following *e* with nonempty $\mathrm {head}(e) \,\cap \, \mathrm {tail}(f)$ (else *e* can be removed from *P*, contradicting minimality); applying this same process again at *f* yields a subsequence of the ordering of *P* that ends in a hyperedge whose head contains *t*; considering this subsequence in reverse order satisfies Definition 4 for backward traceability of *e* from *t*. Hence restricting to the doubly-reachable subgraph ${\widetilde{G}}$ is safe.

To show the implication of the theorem, notice ShortestHyperpathHeuristic explores *all* hyperedges that are forward reachable from *s* in  ${\widetilde{G}}$, inserting hyperedge *e* into heap *H* when *e* is initially reached, again detecting when traversing *e* causes another hyperedge *f* to be first reached using counter *f*.count, and recording in field *f*.inedges all such *e* that have reached *f*. So if an *s*,*t*-hyperpath exists in *G*, which implies sink *t* has an in-edge *e* that is forward reachable from *s* in  ${\widetilde{G}}$, this *e* will eventually be inserted into *H*, making *e*.node non-nil, and at the end of the heuristic causing RecoverShortHyperpath to be called on *e*.

We claim that when function RecoverShortHyperpath (in Fig. 4) is ultimately called on an in-edge to sink *t*, phase (I) first recovers an edge set *S* that is an *s*,*t*-superpath in *G*. Considering the hyperedges of *S* in reverse order of their removal from queue *Q*, they satisfy the three conditions for an *s*,*t*-superpath in Definition 1: the last hyperedge removed from *Q* solely has *s* in its tail, each hyperedge in *S* (other than this last one) has its tail set covered by hyperedges removed later from *Q*, and the first edge removed has *t* in its head.

Function RecoverShortHyperpath in phase (II) then trims *S* to a minimal *s*,*t*-superpath, yielding an *s*,*t*-hyperpath. Finally, ShortestHyperpathHeuristic returns the shortest such hyperpath found.

Thus whenever a source-sink hyperpath exists, the heuristic finds one.${\square}$

Next we prove the heuristic actually solves Shortest Hyperpaths when the input is a singleton-tail hypergraph.

### Optimality of the heuristic for singleton-tail hypergraphs

While our heuristic does not necessarily find shortest hyperpaths in general hypergraphs, we can prove that it does find optimal solutions for the following class of hypergraphs.

A *singleton-tail hypergraph* is a directed hypergraph *G* where every hyperedge *e* in *G* has $\bigl |\mathrm {tail}(e)\bigr | = 1$. (The head sets of hyperedges can be arbitrary.) In other words, in singleton-tail hypergraphs, the tails of hyperedges are single vertices.

At a high level, the optimality argument for singleton-tail hypergraphs first shows that shortest source-sink hyperpaths are composed of shortest *s*,*e*-hyperpaths; then argues that the heuristic’s greedy superpath trimming recovers shortest *s*,*e*-hyperpaths when the hyperedge fields hold shortest hyperpath lengths; and finally proves that the heuristic computes exact shortest *s*,*e*-hyperpath lengths.

The following characterization states that in singleton-tail hypergraphs, a shortest *s*,*t*-hyperpath is composed of shortest *s*,*e*-hyperpaths to its constituent hyperedges. This does not hold for general hypergraphs, and is partly why the special case of shortest singleton-tail hyperpaths is polynomial-time solvable.

#### Lemma 1

**(Characterizing shortest singleton-tail hyperpaths)**  *In singleton-tail hypergraphs with nonnegative edge weights, every shortest* *s*,*t*-*hyperpath can be ordered as a sequence* $e_1 \cdots e_k$ *of hyperedges where*

(i) *each* $$\mathrm {head}(e_i) \,\supseteq \, \mathrm {tail}(e_{i+1})$$*, and*
(ii) *every prefix* $e_1 \cdots e_i$ *is a shortest* $s,e_i$-*hyperpath*.

#### Proof

Consider a shortest *s*,*t*-hyperpath *P* in a singleton-tail hypergraph. By definition, *P* is a minimal *s*,*t*-superpath, so its edges can be ordered as a sequence ${e_1 \cdots e_k}$ where $\mathrm {tail}(e_1) = \{s\}$, ${\mathrm {head}(e_k) \supseteq \{t\}}$, and since tail sets contain a single vertex, for every hyperedge $e_j$ in this sequence other than the first one, there is a prior hyperedge $e_i$ with ${\mathrm {head}(e_i) \,\supseteq \, \mathrm {tail}(e_j)}$.

Starting from the last hyperedge $e_k$, and repeatedly picking a prior hyperedge whose head covers the tail of the current hyperedge until reaching tail $\{s\}$, yields a subsequence $f_1 \cdots f_\ell$ specifying subset ${Q \,=\, \{f_1, \ldots , f_\ell \} \,\subseteq \, P}$, where again ${\mathrm {tail}(f_1) = \{s\}}$, ${\mathrm {head}(f_\ell ) \supseteq \{t\}}$, and now ${\mathrm {head}(f_i) \,\supseteq \, \mathrm {tail}(f_{i+1})}$ for ${1 \!\le \! i \!<\! \ell }$. Furthermore ${Q = P}$, otherwise *P* is not minimal. So subsequence $f_1 \cdots f_\ell$ is exactly sequence $e_1 \cdots e_k$.

Clearly every prefix $e_1 \cdots e_i$ is an $s,e_i$-superpath. Moreover this prefix must be a minimal $s,e_i$-superpath, otherwise  *P* is not minimal. Thus every prefix ending in $e_i$ is an $s,e_i$-hyperpath.

Finally, every prefix $e_1 \cdots e_i$ must be a shortest $s,e_i$-hyperpath. Otherwise, replacing this prefix by a shortest $s,e_i$-hyperpath yields an *s*,*t*-superpath *S* of total weight less than *P*. Furthermore, trimming *S* to a minimal *s*,*t*-superpath under nonnegative edge weights yields an *s*,*t*-hyperpath of total weight less than *P*, contradicting the optimality of *P*.${\square}$

In the following, the *distance* of hyperedge *e* from source *s* is the total weight of a shortest *s*,*e*-hyperpath, which we denote by *d*(*e*). Recall that function ShortestHyperpathHeuristic (in Fig. 3) maintains the field *e*.length, that holds the total weight of the best-known *s*,*e*-hyperpath, which upper bounds *d*(*e*).

The next lemma states that in singleton-tail hypergraphs, given two key conditions, the greedy superpath trimming that is used by the heuristic to recover a hyperpath to hyperedge *e* in fact finds a shortest *s*,*e*-hyperpath.

#### Lemma 2

**(Recovering hyperpaths in singleton-tail hypergraphs)**  *In a singleton-tail hypergraph with nonnegative edge weights, when the hyperpath heuristic recovers a hyperpath from source* *s* *to hyperedge* *e*, *suppose*

(i) *field* *e.inedges contains among its hyperedges an in-edge to* *e* *from a shortest s*,*e-hyperpath, and*
(ii) *in the* *s*,*e-superpath* *S* *found when recovering a hyperpath to* *e*, *for all hyperedges* $f \in S \!-\! \{e\}$, *field* *f*.*length holds distance* *d*(*f*).

*Then the hyperpath to* *e* *that the heuristic recovers is a shortest* *s*,*e-hyperpath.*

#### Proof

We first claim that under the assumptions of the lemma, when the hyperpath heuristic calls RecoverShortHyperpath (in Fig. 4) on a hyperedge *e*, its first phase recovers an *s*,*e*-superpath *S* that contains a shortest *s*,*e*-hyperpath. By assumption (i), field *e*.inedges contains a hyperedge *f* on a shortest *s*,*e*-hyperpath, and *f* will be in superpath *S*, hence by assumption (ii), the value of *f*.length is *d*(*f*). This value came from a shortest *s*,*f*-hyperpath *Q* that was found in a prior call to RecoverShortHyperpath on *f*, by trimming an *s*,*f*-superpath *T*. Notice that *Q* followed by *e* is an *s*,*e*-superpath ${\widetilde{P}}$, as $\mathrm {head}(f) \supseteq \mathrm {tail}(e)$. Now trim ${\widetilde{P}}$ to an *s*,*e*-hyperpath *P*, and let $P^*$ be a shortest *s*,*e*-hyperpath containing *f* that exists by assumption (i). By Lemma 1 and minimality of hyperpaths, $P^*$ must consist of a shortest *s*,*f*-hyperpath $Q^*$ followed by *e*. Under nonnegative edge weights,

$$\begin{aligned} \omega (P) \,\,\le & \,\,\, \omega ({\widetilde{P}}) \\ \,\,= & \,\,\, \omega (Q) + \omega (e) \\ \,\,= & \,\,\, \omega (Q^*) + \omega (e)\\ \,\,= & \,\,\, \omega (P^*) \, . \end{aligned}$$

Thus *P* is also a shortest *s*,*e*-hyperpath. Since *f* is in *e*.inedges, tracing back from *e* recovers the superpath

$$\begin{aligned} S \,\,\,\supseteq \,\,\, T \cup \{e\} \,\,\,\supseteq \,\,\, Q \cup \{e\} \,\,=\,\, {\widetilde{P}} \,\,\supseteq \,\, P \, , \end{aligned}$$

so the claim holds.

We next claim that when RecoverShortHyperpath in its second phase greedily trims superpath *S*, the resulting superpath $T \subseteq S$ still contains a shortest hyperpath. To show this, we prove that each superpath $S_i$ that remains after *i* iterations of greedy trimming contains a shortest *s*,*e*-hyperpath, by induction on *i*. For the basis at $i \!=\! 0$, the initial superpath $S_0$ before any trimming contains a shortest hyperpath by our first claim on *S*. For the induction at $i \!>\! 0$, let *P* be a shortest *s*,*e*-hyperpath that superpath $S_{i-1}$ contains by our hypothesis, and let *f* be the hyperedge removed from $S_{i-1}$ at iteration *i*. If $f \not \in P$, then $S_i \,=\, S_{i-1} - \{f\}$ trivially contains *P*. So we assume $f \in P$. In the following, the *core* of hyperpath *P* consists of the tail vertices of its hyperedges.

In an ordering of shortest hyperpath *P* that satisfies Lemma 1, consider the hyperedges in the suffix of *P* that begins with *f*. As edge weights are nonnegative, by Lemma 1 the distances of these hyperedges must be at least *d*(*f*), so by assumption (ii) the values of the length field for these hyperedges must be at least *f*.length. Greedy trimming proceeds in decreasing order of length-field values, so the hyperedges in this suffix of *P* must either have been already considered for trimming before *f*, or not yet considered due to being tied with *f* (from having zero edge-weight). If they were considered before *f*, then since they were not trimmed, there must be no alternate *s*,*e*-hyperpath in $S_{i-1}$ that enters their head vertices on the core of *P*. If they were not considered yet, then since *f* can be removed from $S_{i-1}$, there must be an alternate *s*,*e*-hyperpath $Q \subseteq S_i$ distinct from *P* that enters one of the core head-vertices of the hyperedges in this suffix of *P* whose length field is tied with *f*. Moreover, this alternate hyperpath *Q* must enter *P* with the same length-field value as the edge of *P* sharing this core head-vertex. (If *Q* enters *P* at a smaller length-value, then *P* is not a shortest *s*,*e*-hyperpath; if *Q* enters at a greater length-value, hyperedge *f* would not be the next hyperedge removed, as instead a hyperedge from *Q* of greater length would be.) Since *Q* enters *P* at the same length-value, hyperpath *Q* is also a shortest *s*,*e*-hyperpath. Hence $S_i \supseteq Q$ still contains a shortest hyperpath, which proves the second claim.

So the final trimmed *s*,*e*-superpath *T* returned by RecoverShortHyperpath contains a shortest *s*,*e*-hyperpath $P \subseteq T$. Since *T* is minimal (as no further edges could be trimmed), and *P* by definition is minimal, we must have $T = P$, which proves the lemma. ${\square}$

We now show that the hyperpath heuristic solves Shortest Hyperpaths for singleton-tail hypergraphs.

#### Theorem 3

**(Optimality of the heuristic on singleton-tail hypergraphs)**  *For singleton-tail hypergraphs with nonnegative edge weights, the hyperpath heuristic finds a shortest source-sink hyperpath. *

#### Proof

The key to proving optimality is showing that in singleton-tail hypergraphs, the estimates that the heuristic computes for shortest hyperpath lengths are exact. Recall that when function ShortestHyperpathHeuristic (in Fig. 3) removes hyperedge *e* from heap *H*, it calls RecoverShortHyperpath on *e* to recover an *s*,*e*-hyperpath *P*, and sets the field $e.\text {length}$ to $\omega (P)$, the total weight of *P*.

We claim that when this assignment occurs, field *e*.length holds distance *d*(*e*), the total weight of a shortest *s*,*e*-hyperpath. We now prove this claim by induction on the number of heap extractions. At a high level, the argument is similar to that for Dijkstra’s shortest-path algorithm (see [26, pp. 659–661]) on ordinary directed graphs.

For the basis, the first hyperedge extracted has $\mathrm {tail}(e) = \{s\}$ and $e.\text {key} = \omega (e)$, which equals *d*(*e*), as *e* itself is a shortest *s*,*e*-hyperpath (since all edge weights are nonnegative). The recovered *s*,*e*-hyperpath will consist of *e* (as *e*.inedges is empty), so after the assignment field *e*.length holds *d*(*e*).

For the induction, let *e* be the next hyperedge to be removed from the heap, and assume for all hyperedges *h* extracted prior to *e* that *h*.length holds *d*(*h*). Now consider a shortest *s*,*e*-hyperpath *P*, and in the ordering of *P* given by Lemma 1, let *f* be the first hyperedge in *P* that has not yet been removed from the heap. Note that *f* exists, as *e* has not been removed yet.

We first show $f.\text {key} = d(f)$. In the special case where *f* is the first edge of *P*, notice $d(f) = \omega (f)$ by the same reasoning as in the basis. Furthermore $f.\text {key} = \omega (f)$, as *f*.key starts at $\omega (f)$, never increases, and cannot decrease below this minimum value. So ${f.\text {key} = d(f)}$ in this special case.

**Fig. 5:**
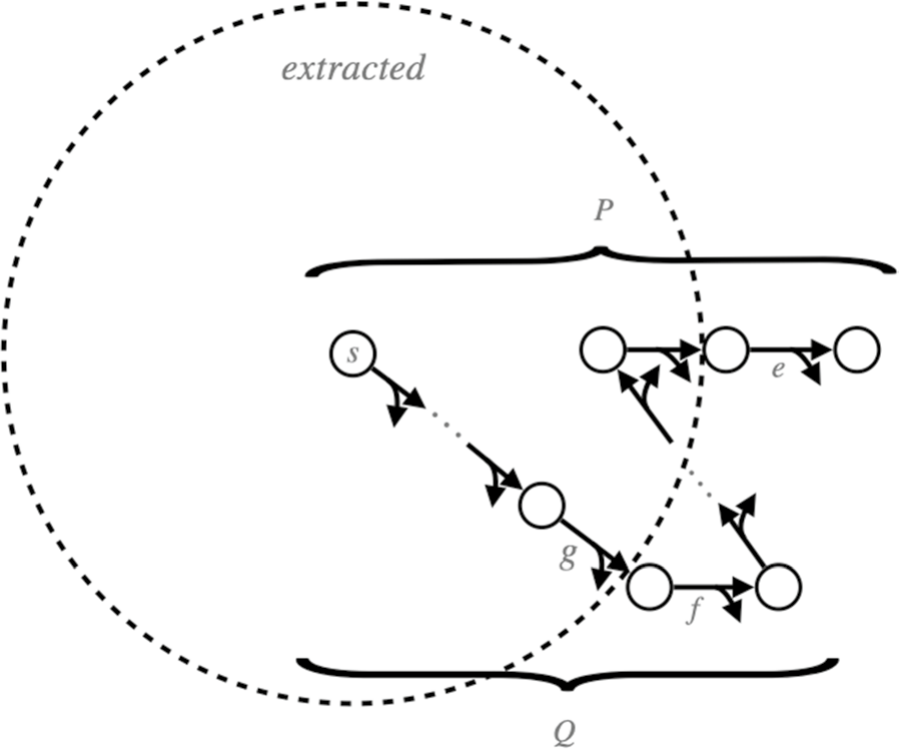
Hyperpath from the proof of optimality for singleton-tail hypergraphs. Hyperedges inside the dashed circle have been extracted from the heap; those outside have not. The next hyperedge to be extracted is *e*, and *P* is a shortest *s*,*e*-hyperpath. The first hyperedge of *P* not yet extracted is *f*, and *Q* is the prefix of *P* up through *f*

In the general case where *f* is not the first edge of *P*, let *g* be the in-edge to *f* on *P*, and $Q \subseteq P$ be the prefix of *P* ending in *f*, as illustrated in Fig. 5. Notice *g* has already been extracted from the heap (by the definition of *f*), so *g* is in *f*.inedges (as when a hyperedge is extracted, for all its out-edges *h* it is added to *h*.inedges). Furthermore *Q* is a shortest *s*,*f*-hyperpath by Lemma 1, so *g* is on a shortest hyperpath to *f*. For all hyperedges *h* extracted before *e*, by the induction hypothesis $h.\text {length} = d(h)$, and only extracted *h* add themselves to the field inedges of their out-edges. Hence when *g* was extracted, added itself to *f*.inedges, and updated *f*.key by recovering an *s*,*f*-hyperpath, in the *s*,*f*-superpath *S* first found during recovery, all hyperedges $h \in S$ had $h.\text {length} = d(h)$. Thus by Lemma 2, the recovered *s*,*f*-hyperpath was a shortest hyperpath, so this updated *f*.key to *d*(*f*), and as argued before in the special case, this key will not change. So again $f.\text {key} = d(f)$.

We next show,

1 $$\begin{aligned} e.\text {key}\,\le \,\,\,& {} f.\,\text {key} \end{aligned}$$

2 $$\begin{aligned}=\,\,\, & {} d(f) \end{aligned}$$

3 $$\begin{aligned}\le\,\,\, & {} d(e) \end{aligned}$$

4 $$\begin{aligned}\le\,\,\, & {} e.\text {key} \, \,. \end{aligned}$$

In the above, inequality (1) holds since *e* and *f* are both on the heap (as *f* was inserted in the heap either during initialization or when *g* was extracted), but *e* is removed before *f*. Equation (2) is from our prior analysis of *f*. Inequality (3) holds as *Q* and *P* are shortest *s*,*f*- and *s*,*e*-hyperpaths respectively, while ${Q \subseteq P}$ and edge weights are nonnegative. Lastly, inequality (4) holds since the key of *e* while it is on the heap is the total weight of some *s*,*e*-hyperpath. Thus relations (1)–(4) must all be equalities, so $e.\text {key} = d(e)$.

We now argue $e.\text {length} = d(e)$ after *e* is extracted. Since $e.\text {key} = d(e)$ is the weight of a hyperpath recovered earlier for *e*, notice (i) there was an in-edge to *e* on a shortest *s*,*e*-hyperpath in *e*.inedges; moreover (ii) all hyperedges *h* in the *s*,*e*-superpath collected while recovering a hyperpath for *e* were extracted earlier, and hence by the induction hypothesis had $h.\text {length} = d(h)$. Furthermore, hyperedges are never removed from the field inedges, and *h*.length never changes after *h* is extracted. Thus the assumptions in Lemma 2 are still met upon extraction of *e*, so when ShortestHyperpathHeuristic assigns to *e*.length the total weight of the hyperpath *P* recovered for *e*, by Lemma 2 this recovered *P* will again be a shortest *s*,*e*-hyperpath, hence $e.\text {length} = d(e)$. This completes the inductive proof of our claim.

So for every hyperedge *h* in the doubly-reachable subgraph explored by ShortestHyperpathHeuristic, after extracting *h* from the heap, the relation $h.\text {length} = d(h)$ holds. Finally, when recovering the best *s*,*t*-hyperpath at the end of the heuristic by examining the in-edges *e* to sink *t*, for each such hyperedge *e* the assumptions of Lemma 2 are still met (by the same reasoning as above), so the hyperpaths *P* obtained from calling RecoverShortHyperpath on these sink in-edges *e* are again shortest *s*,*e*-hyperpaths. Since a shortest *s*,*t*-hyperpath consists of doubly-reachable hyperedges (by the proof of Theorem 2), and is a shortest *s*,*e*-hyperpath for some in-edge *e* to sink *t*, the best of these recovered hyperpaths *P*, which is the hyperpath returned by the heuristic, is a shortest *s*,*t*-hyperpath.${\square}$

Theorem 3 (in combination with Theorem 1) shows that, while Shortest Hyperpaths is NP-complete for singleton-*head* hypergraphs [14], it is polynomial-time solvable for singleton-*tail* hypergraphs.

## Generating all source-sink hyperpaths

In this section, we give a practical algorithm for generating *all* *s*,*t*-hyperpaths in a given hypergraph for a fixed source *s* and sink *t*. In our later experimental results, we use this algorithm on specific source-sink instances from real cell-signaling networks to tractably measure how close our heuristic is to optimal.

In general, the technique of inclusion and exclusion of Hamacher and Queyranne [27] provides a widely-applicable method for generating all the solutions to any combinatorial optimization problem whose feasible solutions are subsets of a ground set—where in our context, hyperpaths are subsets of hyperedges from a hypergraph—but it relies on the ability to efficiently compute a feasible solution that is constrained to include a given *in-set* and exclude a given *out-set*. Interestingly, for hyperpaths, Carbonell et al. [20] have shown that just determining whether an *s*,*t*-hyperpath exists that contains a specified in-set of hyperedges (regardless of the length of the hyperpath) is already NP-complete. Consequently, we cannot generate all *s*,*t*-hyperpaths using the standard inclusion-exclusion technique, as we cannot tractably solve the resulting subproblem that has both in- and out-set constraints.

**Fig. 6:**
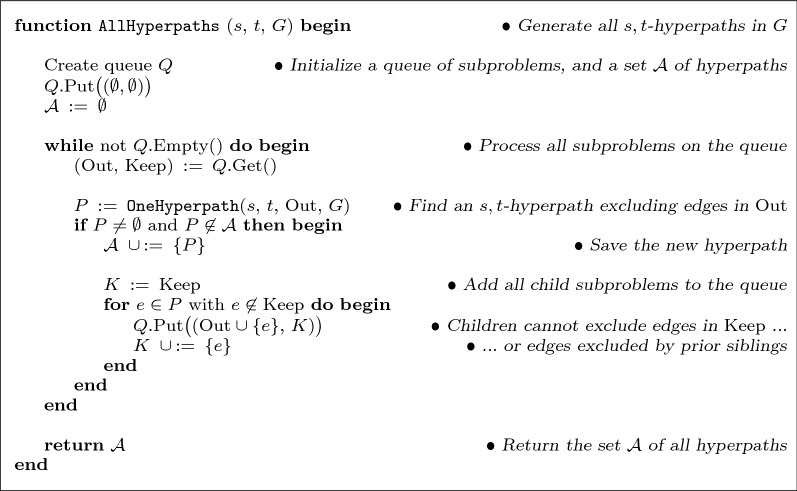
Generating all source-sink hyperpaths. Function AllHyperpaths, given source vertex *s*, sink vertex *t*, and hypergraph *G*, returns the set of all *s*,*t*-hyperpaths in *G*. It calls a function OneHyperpath that returns an *s*,*t*-hyperpath not containing any hyperedge from a specified set Out, and which returns the empty path if no such hyperpath exists

Instead, we generate all hyperpaths through a simple and practical algorithm that only involves out-sets, given in Fig. 6. Function AllHyperpaths returns a list of all *s*,*t*-hyperpaths in hypergraph *G*, leveraging a function OneHyperpath that just has to return one *s*,*t*-hyperpath *P* in *G* that does not contain any hyperedges from set Out (so $P \cap \text {Out} = \emptyset$), or determine that no such hyperpath exists. This constrained hyperpath problem with only out-sets is easy to solve: remove all hyperedges in set Out from *G*, collect all vertices *R* and hyperedges *F* reachable from *s* in this reduced hypergraph, and if $t \in R$, then find any minimal subset $P \subseteq F$ in which *t* is still reachable from *s*; otherwise if $t \not \in R$, no such hyperpath exists. Function OneHyperpath can efficiently find such an *s*,*t*-hyperpath *P* excluding set Out using repeated calls to ForwardReachable (given earlier in Fig. 2).

Function AllHyperpaths uses a queue of subproblems. A subproblem is described by a pair $(\text {Out}, \text {Keep})$, which corresponds to finding an *s*,*t*-hyperpath excluding Out, where any subsequent subproblems that arise from this given subproblem must not exclude any hyperedges from the set Keep (though their solutions are not required to actually use edges from Keep). The purpose of this set Keep is to ensure that all subproblems ever placed on the queue have distinct Out sets. (So any given subproblem described by an out-set is only ever solved once, as argued in the later section on the time complexity of the hyperpath enumeration algorithm in the proof of Theorem 5.) A subproblem that directly arises from a given one we call a *child* subproblem (as the entire collection of subproblems conceptually forms a tree that is explored breadth-first using the queue). Each child subproblem excludes one edge from the hyperpath found for its parent subproblem; in this way, the children will generate hyperpaths that are distinct from their parent hyperpath, if they have a solution. (Once a subproblem becomes infeasible due to its out-set eliminating any *s*,*t*-hyperpath as a solution, it also does not generate further subproblems.) Though the whole approach never repeatedly solves the same subproblem, in contrast to the inclusion-exclusion technique it can generate the same hyperpath from different subproblems, so we check whether hyperpath *P* is distinct from those already found before adding it to the list ${\mathcal {A}}$ of all hyperpaths.

We first prove this enumeration approach is correct, and then analyze its time complexity.

### Correctness of the hyperpath enumeration algorithm

We next show that function AllHyperpaths solves the problem of source-sink hyperpath enumeration.

#### Theorem 4

**(Correctness of hyperpath enumeration)**  *The hyperpath enumeration algorithm generates every* *s*,*t*-*hyperpath exactly once. *

#### Proof

For the function AllHyperpaths (in Fig. 6), we view the subproblems it processes as forming a tree: when a problem *p* is pulled off queue *Q* and causes a new subproblem *q* to be put onto *Q*, these subproblems *q* comprise the children of *p* in the tree. Each subproblem is specified by a pair $(\text {Out}, \text {Keep})$, representing the problem of finding an *s*,*t*-hyperpath that contains no hyperedge in the set Out. Let *P* be an *s*,*t*-hyperpath satisfying this out-constraint for problem *p*. Any other *s*,*t*-hyperpath ${\widetilde{P}}$ distinct from *P* that also satisfies the out-constraint for *p* must not contain some hyperedge in *P*. (If ${\widetilde{P}}$ contains every hyperedge of *P* yet is distinct, it is a strict superset of *P*, contradicting minimality.) Function AllHyperpaths forms the children of *p* by adding each hyperedge in *P* to the out-set of *p* for a different child. (So the hyperpaths satisfying the out-constraints of the children are all hyperpaths that both satisfy the constraints of parent *p* and are distinct from hyperpath *P*.) Consequently hyperpath *P*, together with every solution to the children of *p*, comprise all possible solutions to problem *p*.

This tree-like process begins at the root with a problem having an empty out-set (whose solutions are all possible *s*,*t*-hyperpaths), and continues refining each problem into its children subproblems until reaching the leaves (which have no solution). Thus the set consisting of each hyperpath *P* found at the nodes of this tree contains all *s*,*t*-hyperpaths.

In brief, function AllHyperpaths generates every *s*,*t*-hyperpath. Since it checks for uniqueness, the enumeration algorithm generates every source-sink hyperpath exactly once.${\square}$

### Time complexity of the hyperpath enumeration algorithm

We now bound the running time of function AllHyperpaths in terms of the number of subproblems it solves, and parameters of the input hypergraph.

#### Theorem 5

**(Time complexity of hyperpath enumeration)**  *The running time of the hyperpath enumeration algorithm, when solving* *k* *subproblems on a hypergraph of size* $\ell$ *with* *m* *hyperedges, is*

$$\begin{aligned} O\bigl ( k \, \ell \, m \bigr ) \,\,\,=\,\,\, O\bigl ( 2^m\, \ell \, m \bigr ) \, . \end{aligned}$$

#### Proof

We bound the running time of function AllHyperpaths (in Fig. 6) as follows. Solving a given subproblem from the queue by function OneHyperpath (which finds an *s*,*t*-hyperpath by iteratively removing hyperedges from the hypergraph and testing reachability to identify a minimal set in which *t* is still reachable from *s*), involves at most *m* calls to function ForwardReachable. A call to ForwardReachable takes $O(\ell )$ time (by the analysis in the proof of Theorem 1), so solving a subproblem takes $O(\ell \, m)$ time. If AllHyperpaths terminates after processing *k* subproblems, its total time is then $O(k \, \ell \, m)$.

We argue next that the out-sets of subproblems are all distinct. Consider the tree of subproblems processed by AllHyperpaths (as in the proof of Theorem 4), and two arbitrary subproblems *x* and *y* in this tree. If one of *x* and *y* is a descendant of the other, their out-sets are distinct, as a child always adds a hyperedge to the out-set of its parent. If neither *x* nor *y* is a descendant of the other, let subproblem *u* be their nearest common ancestor, subproblems *v* and *w* be the children of *u* on the paths to *x* and *y* respectively, and assume without loss of generality that child *v* precedes child *w*. When child *v* adds hyperedge *e* to the set Out of its parent *u*, edge *e* is not added to set Out for any other children of *u*, and *e* is also added to set Keep for all children of *u* following *v*, including *w*. Furthermore, the set Out for a descendant is a superset of set Out for its ancestors, and set Out for a descendant is always disjoint from set Keep for its ancestors. Consequently, the above hyperedge *e* is in the out-set of subproblem *x* but not subproblem *y*, so their out-sets are again distinct.

Since subproblem out-sets are distinct, $k \,=\, O(2^m)$. Combining this with the prior total time for hyperpath enumeration yields a worst-case time bound of $O(2^m \, \ell \, m)$.${\square}$

In practice, typically $k \ll 2^m$, so the running time is much faster than the worst-case bound suggests. Function AllHyperpaths can tractably generate all source-sink hyperpaths for large hypergraphs, as shown in the next section on experimental results, since many of its subproblems quickly become infeasible for real cell-signaling networks.

## Experimental results

We now present results from computational experiments on real pathway databases that compare the hyperpath found by our heuristic to the optimal solution. We also remark on the prevalence of biological instances with cyclic shortest hyperpaths, study the cause of suboptimality in our heuristic, report actual running times, and discuss biological examples of cyclic hyperpaths.

### Datasets

We evaluate the quality of our heuristic on four datasets built by combining different annotated signaling pathways from two pathway databases, NCI-PID and Reactome. NCI-PID [28] is a curated human-pathway database containing biochemical reactions for complex assembly, cellular transport, and transcriptional regulation. Reactome [29] also contains curated human signaling pathways, and is actively maintained with new reactions being continuously added. We constructed hypergraphs from three subsets of NCI-PID pathways used in Ritz et al. [5], named the Small, Medium, and Large datasets. The Small dataset is a small Wnt signaling pathway consisting of the union of two pathways: “degradation of $\beta$-catenin” and “canonical Wnt signaling”. The Medium dataset is a larger Wnt signaling pathway including four additional pathways: “noncanonical Wnt signaling”, “Wnt signaling network”, “regulation of nuclear $\beta$-catenin”, and “presenilin action in Notch and Wnt signaling”, which correspond to non-canonical branches of Wnt signaling. The Large dataset contains all NCI-PID pathways. Similarly, the Reactome dataset is the union of all Reactome pathways. The NCI-PID and Reactome pathways were downloaded in the BioPAX format [30] from Pathway Commons, and processed using a parser from Franzese et al. [22] built on PaxTools [31].

To construct the hypergraphs for each dataset, we mapped each entity (such as a protein, small molecule, and so on) to a vertex in the hypergraph. Each complex was represented as a unique vertex distinct from the entities in the complex. Multiple forms of the same protein map to different vertices denoting compartmentalization and post-translational modifications, such as phosphorylation and ubiquitination. We treated each variant as a distinct entity because many pathways describe the transportation of a protein from one cellular compartment to another, or the marking of a protein for degradation by ubiquitination, necessitating that the corresponding vertices be distinct to reflect these variants. Each reaction was mapped to a hyperedge, where the reactants and positive regulators comprise the tail of the hyperedge, and the products comprise the head. All hyperedges were given unit weight, even though the heuristic handles weighted edges, as NCI-PID is missing reaction rates for some reactions.

**Table 1:** Dataset Summaries

	NCI-PID							
Measure	Small		Medium		Large		Reactome	
Vertices	56		350		9,009		20,458	
Hyperedges	36		228		8,456		11,802	
Pathways	2		6		213		2,516	
Sources	19		138		3,200		8,296	
Targets	10		102		2,636		5,066	
Self-loops	1		8		40		433	
Unreachable self-loops	1		7		14		32	

	mean	max	mean	max	mean	max	mean	max
Tail size	1.8	3	1.9	5	1.9	10	2.4	26
Head size	1.3	3	1.3	4	1.1	5	1.6	28
Forward-reachable set	35	35	192	192	6,169	6,169	4,645	4,645
Backward-traceable set	28	28	49	70	1,198	2,863	4,027	7,021
Doubly-reachable set	27	27	42	60	756	1,836	929	1,725
In-degree	0.8	5	0.8	15	1.0	323	0.9	1,056
Out-degree	1.1	4	1.2	24	1.7	326	1.4	1,167

Table 1 gives statistics on the hypergraphs constructed from each of the four datasets. The hypergraphs are very sparse: there are fewer hyperedges than vertices in all four datasets, with Reactome being even sparser than the NCI-PID datasets. The hypergraphs from the Large and Reactome datasets contain respectively 40 and 433 self-loops, showing that many cyclic hyperpaths are likely to exist. However, a small number of these self-loops are unreachable, due to an otherwise unreachable vertex appearing in both the head and tail of the hyperedge. The sources and targets used in all our experiments are respectively vertices with no in-edges (or vertices whose only in-edge is an unreachable self-loop), and vertices with no out-edges. The number of forward-reachable, backward-traceable, and doubly-reachable hyperedges shows how many hyperedges remain after the heuristic prunes the input hypergraph to the doubly-reachable subgraph before computing a solution. On average, hyperedges from all four hypergraphs have small head and tail sets, and vertices have low in- and out-degree, reflecting the sparseness of the hypergraphs.

### Experimental setup

To prepare the hypergraphs from each dataset for our experiments, we parsed the union of the pathways in the dataset. We connected a *supersource* *s* to all *source* vertices—namely, the input vertices with no in-edges—by a single zero-weight hyperedge whose tail consisted of the supersource *s* and whose head contained all the source vertices. We also included in the head of this hyperedge from supersource *s* all input vertices whose sole in-edge was a self-loop, since otherwise such a self-loop was not traversable. For each specific *target* vertex *v*—namely, each input vertex with no out-edges—we had a separate version of the hypergraph that differed only by connecting this target *v* to a *sink* *t* by a single zero-weight ordinary-graph edge directed from *v* to *t*, giving us a specific target instance. Note that these choices for the source and target vertices are reasonable, as they are the molecules where biologists stopped annotating a given pathway. Note also that the supersource *s* and the sink *t* remain the same across all target instances in a dataset.

For each target instance, we trimmed the hypergraph to the doubly-reachable set: the set of hyperedges that were both forward-reachable from supersource *s*, and backward-traceable from sink *t*. Table 1 gives the average and maximum size of the forward-reachable, backward-traceable, and doubly-reachable sets over all target instances for a given dataset, which dramatically reduces the size of the hypergraph over which the heuristic performs most of its computation.

For each target instance, we found a hyperpath from supersource *s* to sink *t* using our shortest hyperpath heuristic implemented in the new tool Hhugin [25], and compared its length to the solution of the MILP of Ritz et al. [21] if the heuristic hyperpath was acyclic. For each cyclic target instance where the heuristic output a cyclic hyperpath, we exhaustively enumerated all *s*,*t*-hyperpaths, and compared the heuristic hyperpath to the shortest hyperpath found by this enumeration. (Enumerating all *s*,*t*-hyperpaths for one source-sink instance takes on average around 20 hours in practice—so it is not feasible to perform this enumeration on all acyclic target instances.)

### Abundance of cyclic hyperpaths

Cyclic shortest hyperpaths appear in all four datasets. To take just one example, in the Small and Medium datasets, the only hyperpath from ubiquitinated $\beta$-catenin to APC is cyclic, so for this target instance the acyclic shortest-hyperpath MILP fails to find a solution. Admittedly this particular source-target pair is specially chosen, as ubiquitinated $\beta$-catenin has an in-edge and APC has an out-edge so they would not normally be considered under our definition of sources and targets. Nevertheless, this pair demonstrates there do exist cyclic hyperpaths in the NCI-PID database—even in the union of just two pathways—that are missed by the current state-of-the-art when computing only acyclic shortest hyperpaths.

In the Large dataset, 38 target instances have cyclic heuristic hyperpaths. Of these, 22 were cyclic because of a self-loop, and 16 were cyclic due to a non-trivial cycle. For all these instances, no acyclic hyperpath exists between supersource *s* and sink *t*. It is likely that even more cycles exist within the hypergraph from the Large dataset, as there were 8 self-loops that were not on any hyperpath found by the heuristic.

In the Reactome dataset, the heuristic found a cyclic shortest hyperpath on 22 target instances, and only one of these instances was cyclic due to a self-loop. In general, Reactome is much sparser than NCI-PID, and 432 of the 433 self-loops in Reactome are never used in a heuristic hyperpath.

The abundance of cyclic hyperpaths in the NCI-PID and Reactome datasets demonstrates the importance of a shortest hyperpath algorithm that properly handles cycles. We discuss concrete examples of biological cyclic shortest hyperpaths in a later section on biological examples.

**Table 2:** Acyclic Instance Summaries

	NCI-PID			
Measure	Small	Medium	Large	Reactome
Target instances	10	102	2,636	5,066
Reachable instances	10	90	2,220	2,432
Acyclic instances	9	89	2,182	2,410
Heuristic was optimal	100% (9)	100% (89)	99% (2,159)	100% (2,410)

### Quality of the hyperpath heuristic

To determine the quality of our hyperpath heuristic, we compared the length of the heuristic hyperpath to an optimal shortest hyperpath. In general, no practical exact algorithm is currently known for finding a shortest source-sink hyperpath. Consequently, on the target instances where the heuristic found a *cyclic* hyperpath, we determined the optimum by generating all source-sink hyperpaths and retaining the shortest one, using our algorithm for hyperpath enumeration. On the target instances where the heuristic found an *acyclic* hyperpath, we compared its length just to the optimal hyperpath returned by the MILP for shortest acyclic hyperpaths. An even shorter cyclic hyperpath could exist for these latter instances, but finding it by enumerating all hyperpaths is simply too time-consuming to carry out for every such instance.

**Table 3:** Cyclic Instance Summaries

Measure	NCI-PID							
	Small		Medium		Large		Reactome	
Target instances	10		102		2,636		5,066	
Reachable instances	10		90		2,220		2,432	
Cyclic instances	1		1		38		22	
Heuristic was optimal	100%		100%		100%		100%	
Non-trivial cycles	1		1		22		21	

	median	max	median	max	median	max	median	max
Number of hyperpaths$^*$	1	1	1	1	3	364	22	136
Path length range$^{\dagger }$	0	0	0	0	1	43	[2,3]	15

$^*$Total number of hyperpaths for a cyclic target instance

$^{\dagger }$Difference between the length of the longest and shortest hyperpaths

Table 2 summarizes the quality of the heuristic on acyclic instances. On the Small, Medium, and Reactome datasets, the heuristic hyperpath is optimal on all target instances, meaning the heuristic hyperpath and the shortest acyclic hyperpath from the MILP have the same length. On the Large dataset, the heuristic is optimal on over 99% of the instances, demonstrating the quality of the heuristic on these biological datasets. The small fraction of instances where our heuristic was suboptimal are discussed in more detail in the next subsection.

Table 3 summarizes the quality of the heuristic on instances where it output a cyclic hyperpath. On all these cyclic instances, the acyclic MILP failed to find a solution, so we could not compare the heuristic to an optimal hyperpath other than by exhaustively enumerating all hyperpaths and picking the shortest one—which verified that the heuristic on these instances in fact found an optimal solution. Cyclic instances from the Reactome (and Large) datasets contain many distinct hyperpaths, with a median of 22 (respectively 3) hyperpaths, and a maximum of 136 (respectively 364) hyperpaths. The hyperpaths tend to vary in length, with a maximum difference between the length of the longest and shortest hyperpath of 15 (respectively 43) hyperedges, and a median difference of [2, 3] (respectively 1) hyperedges. This demonstrates that the heuristic is discriminating between hyperpaths of different lengths and choosing the best hyperpath over worse hyperpaths, further indicating the quality of the heuristic. In every cyclic target instance, all *s*,*t*-hyperpaths were cyclic, and many shared a common cycle; most of the hyperedges occurring in one hyperpath but not another appeared outside this shared cycle.

### Studying the suboptimality of the heuristic

We call the small number of target instances in these experiments where the heuristic found a known suboptimal hyperpath its *suboptimal instances*. Table 4 summarizes these 23 suboptimal instances, which are all from the Large NCI-PID dataset, and are all acyclic instances. (The heuristic was optimal on all cyclic instances, and all Reactome, Small, and Medium instances. We mention as well that the maximum values across the table occur in distinct target instances.) To gain insight into why the heuristic found a suboptimal solution on these instances, we enumerated all source-sink hyperpaths for every suboptimal instance. (This enumeration also verified that on all suboptimal instances, the acyclic MILP in fact found a shortest hyperpath, as there was no shorter cyclic hyperpath.)

Hyperpath enumeration confirmed that these suboptimal instances are much harder than the cyclic instances. The median number of hyperpaths is nearly 140 times higher for suboptimal NCI-PID instances compared to cyclic NCI-PID instances, and the length difference between the longest and shortest hyperpaths is 30 times larger. This stark contrast indicates the inherent difficulty of these suboptimal instances, where the heuristic must now discriminate among a much higher number of hyperpaths that have much greater path-length variance. The fraction of all hyperpaths that are optimal is fairly small, with only around 3% being optimal for the median instance. Even faced with many alternate solutions, the heuristic still found a hyperpath that was nearly optimal: the median difference between the length of the heuristic hyperpath and the shortest hyperpath was 1 hyperedge, the maximum difference was 6 hyperedges, and the median ratio of the length of the heuristic hyperpath to the shortest hyperpath was 1.1 (so it was only 10% longer). Next we investigate what could be causing this suboptimality.

The suboptimality of the heuristic is likely coming from the repeated calls to the function RecoverShortHyperpath, which proceeds in two phases. In phase (I), this function *recovers* an *s*,*e*-superpath *S*, relying on in-edge lists to hyperedges *f*, where the in-edge list for *f* contains only hyperedges removed from the heap prior to *f*, which may exclude hyperedges in a shortest *s*,*e*-hyperpath. In phase (II), this function *trims* superpath *S* to a hyperpath by greedily considering hyperedges in *S* for removal, which may also remove a hyperedge in an optimal *s*,*e*-hyperpath.

To determine whether the recover or trim phases were responsible for suboptimality, we ran the following experiment. After the heuristic determined its estimated path length for every hyperedge in the hypergraph, we called RecoverShortHyperpath on each in-edge to the target where we ran its recovery phase but stopped before its trimming phase, and unioned together the resulting *s*,*t*-superpaths from each in-edge to create one large *s*,*t*-superpath *F*. We then took an optimal *s*,*t*-hyperpath *P* and examined whether $P \subseteq F$: in other words, whether the recovery phase permitted the heuristic to potentially find an optimal hyperpath. We discovered that for all 23 suboptimal instances $P \not \subseteq F$, indicating phase (I) of RecoverShortHyperpath that recovers an *s*,*e*-superpath was forcing the heuristic to be suboptimal on every instance.

On the other hand the trimming phase of RecoverShortHyperpath could also be leading to suboptimality, which we investigated as follows. For each suboptimal instance, we modified the recovery phase of RecoverShortHyperpath to use all in-edges in the hypergraph to each hyperedge, rather than the in-edge lists collected by the heuristic. (In this situation, the recovered superpath *F* definitely contains a shortest hyperpath *P*.) Phase (II) then trimmed this superpath as normal. We discovered that the trimming phase often fails to find a shortest hyperpath within this larger superpath (which was the entire doubly-reachable subgraph). This indicates that while phase (I) is definitely causing suboptimality, simply changing phase (I) to recover a larger superpath may in turn lead to suboptimality in phase (II).

**Table 4:**
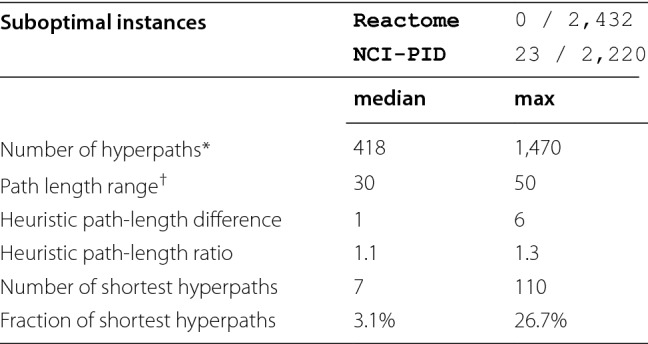
Suboptimal Instance Summaries

$^*$Total number of hyperpaths for a target instance

$^{\dagger }$Difference between the length of the longest and shortest hyperpaths

### Implementation and running time

The heuristic is implemented in Python 2.7.3, comprising around 500 lines of code. The parser used to convert the BioPAX format into hypergraphs is from [22]. For directed hypergraph representation and reachability we used Halp (github.com/Murali-group/halp/). All heuristic and hyperpath enumeration source code is available at http://hhugin.cs.arizona.edu.

Experiments were run on a laptop with a 2.9 GHz Intel Core i5 CPU, and 16 GB of RAM. The running time of the hyperpath heuristic was 55 seconds on average for the instances from the Large and Reactome datasets, which have just under 1000 doubly-reachable hyperedges on average. Memory usage was low, with the heuristic using less than 2 GB of memory.

Enumerating all hyperpaths for the instances is time-consuming, taking 20.4 hours on average for the suboptimal instances with a maximum time of 53.8 hours, which is not practical to carry out for all 4600 target instances.

### Biological examples

**Fig. 7:**
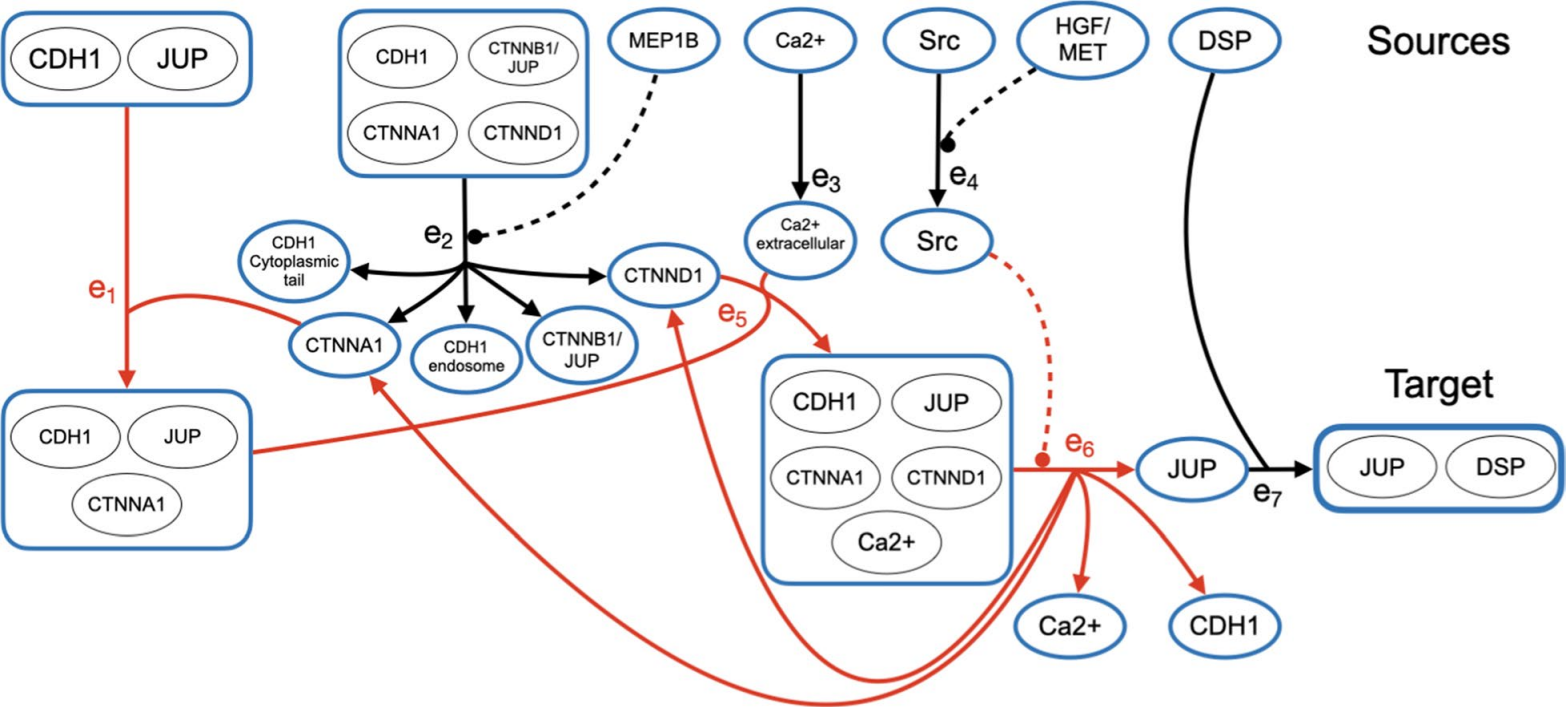
Cyclic shortest hyperpath to the JUP/DSP complex in the Large dataset. All vertices in the hyperpath connected to the supersource are shown at the top of the figure. The hyperedges in this hyperpath come from four different pathways, and show the different complexes JUP participates in until finally being free to bind with desmoplakin (DSP). Positive regulators of reactions are shown by dashed lines ending in a disc. Hyperedges $e_1$, $e_5$, and $e_6$, shown in red, create two separate cycles back to $\alpha$-catenin and $\delta$-catenin

**Fig. 8:**
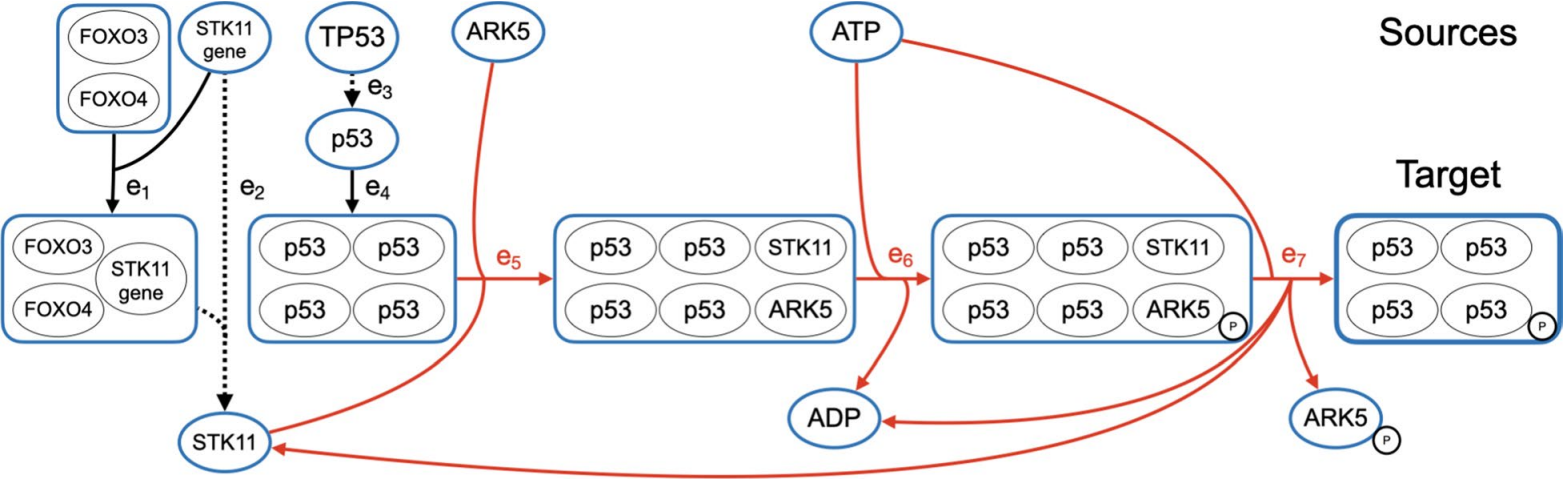
Cyclic shortest hyperpath to phosphorylated p53 in the Reactome dataset. All vertices in the hyperpath connected to the supersource are shown at the top of the figure. The hyperedges in this hyperpath show the transcription of STK11 and p53 (TP53) before NUAK1 (ARK5) participates in the phosphorylation of the p53 tetramer. Hyperedges $e_5$, $e_6$, and $e_7$, shown in red, create a cycle when the phosphorylation of p53 breaks up a complex, returning STK11 to its solitary state. Hyperedges $e_2$ and $e_3$ show transcription, and are drawn dotted

**Fig. 9:**
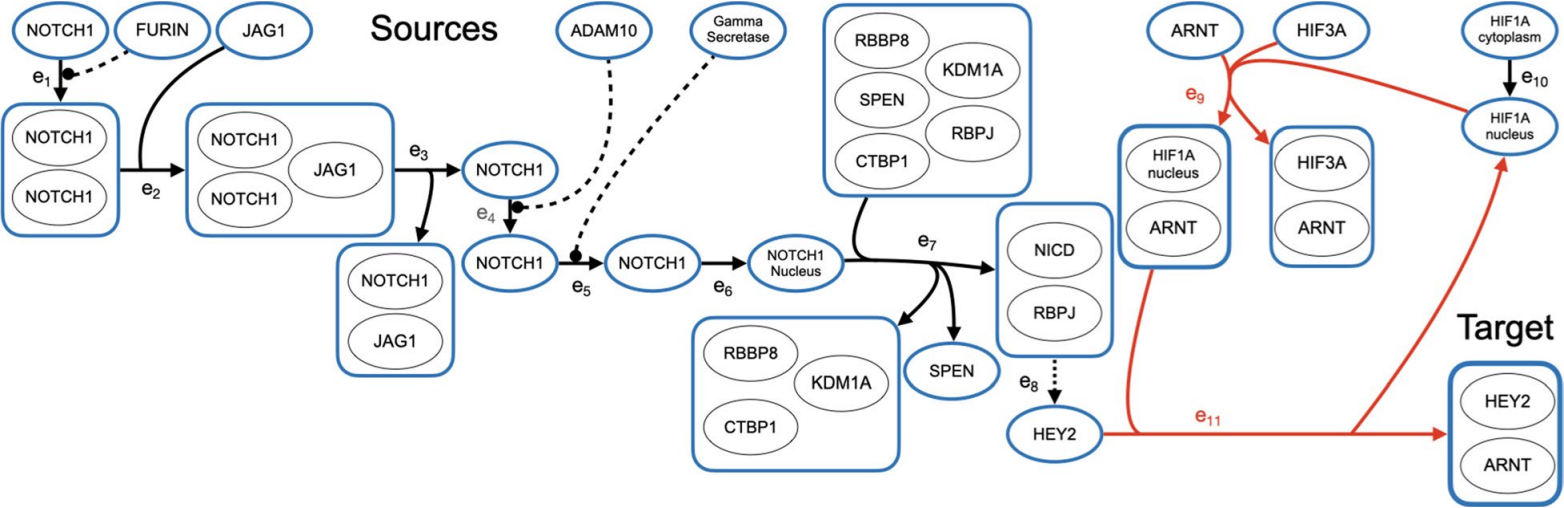
Cyclic shortest hyperpath to the HEY2/ARNT complex in the Large dataset. All vertices from the hyperpath connected to the supersource are shown at the top of the figure. Positive regulators of reactions are shown by dashed lines ending in a disc. The eleven hyperedges span three different NCI-PID pathways, and show the events upstream of HEY2 transcription, ultimately culminating in its repression of ARNT. The cycle between hyperedges $e_9$ and $e_{11}$, shown in red in the figure, recreates nuclear HIF1A. Edge $e_8$, shown dotted, is a template reaction, where the NOTCH1/RBPJ complex upregulates the transcription of the protein HEY2

We now discuss three instances with cyclic shortest hyperpaths from the Large and Reactome datasets. The hyperpath found by our heuristic for these three instances is optimal (as was the case for all instances where the heuristic found a cyclic path), and is drawn in Figs. 7, 8, and 9. We describe the hypergraph structure and constituent reactions for each instance.

*Assembly of the JUP/DSP complex* The first example captures the assembly of the JUP/DSP complex from the Large dataset. Figure 7 shows the shortest hyperpath returned by our heuristic with the JUP/DSP complex as the target. All vertices at the top of the figure are connected to the supersource.

This hyperpath includes seven hyperedges from four different NCI-PID pathways: “E-cadherin signaling in the nascent adherens junction” (hyperedges $e_1$ and $e_5$), “Posttranslational regulation of adherens junction stability and dissassembly” (hyperedges $e_2$, $e_6$ and $e_7$), “Signaling events mediated by PRL” (hyperedge $e_3$), and “Signaling events mediated by hepatocyte growth factor receptor (c-Met)” (hyperedge $e_4$). We briefly describe the key events in this hyperpath. Protein $\gamma$-catenin (also known as junction plakoglobin or JUP) is initially complexed with Cadherin 1 (CDH1) in the tail of hyperedge $e_1$. In hyperedge $e_2$, the metalloprotease meprin$\beta$ cleaves E-cadherin (CDH1), releasing it from its complex with $\alpha$-catenin (CTNNA1) and $\delta$-catenin (CTNND1) [32]. The CDH1/JUP complex adds $\alpha$-catenin (CTNNA1 in hyperedge $e_1$) and CTNND1 and Ca$^{2+}$ (in hyperedge $e_5$) to form a five-member complex. Hepatocyte growth factor (HGF) activates the proto-oncogene tyrosine-protein kinase Src (hyperedge $e_4$) [33]. Src regulates the breakup of this complex into its individual components [34] (hyperedge $e_6$), freeing JUP to bind with DSP and creating the two cycles in this hyperpath via CTNNA1 and CTNNB1. The hyperpath culminates in the formation of a complex between desmoplasmin (DSP) and JUP.

The hypergraph for this instance is large, with 6168 forward-reachable hyperedges, 2642 backward-traceable hyperedges, and 1665 doubly-reachable hyperedges. There is no acyclic hyperpath from the supersource to JUP/DSP. When enumerating all *s*,*t*-hyperpaths for this instance, there were 16 alternate hyperpaths, and the longest hyperpath had 3 more hyperedges than the heuristic path, which was verified to be optimal.

*Phosphorylation of p53*  The second example captures the phosphorylation of p53 by NUAK1 (ARK5) from the Reactome dataset. The heuristic hyperpath, which is optimal, is shown in Fig. 8. All of the vertices at the top are connected to the supersource.

Hyperedge $e_1$ shows the complex formation of FOXO3 and FOXO4 with the STK11 gene, allowing for the transcription of the gene in hyperedge $e_2$. Hyperedges $e_3$ and $e_4$ deal with the transcription of protein p53 (TP53), and its formation into a homotetramer. The p53 tetramer then forms a complex with NUAK1 (ARK5) and STK11 in hyperedge $e_5$, allowing for the phosphorylation of NUAK1 via ATP in hyperedge $e_6$. Once NUAK1 is phosphorylated, it directly phosphorylates p53 [35], activating it and allowing it to assist in DNA damage repair. The final hyperedge $e_7$, shown in red, breaks apart the p53 tetramer/NUAK1/STK11 complex, resulting in a cycle of free STK11. This hyperpath features two transcriptional hyperedges $e_2$ and $e_3$, shown dotted.

This example from Reactome is slightly smaller than the example from the Large dataset, with only 4645 forward-reachable edges, 7021 backward-traceable edges, and 1632 hyperedges in the doubly-reachable set. There was no acyclic hyperpath for this instance. In contrast to the first example, no alternate hyperpaths to the target exist in the hypergraph.

*HEY2/ARNT complex assembly* The final example we discuss is the formation of the HEY2/ARNT complex from the Large dataset. The shortest hyperpath from the supersource to HEY2/ARNT, which was found by the heuristic, is shown in Fig. 9. Once again, the sources are at the top of the figure, with the hyperedge from the supersource not shown.

This hyperpath with eleven edges spans three pathways: “Notch signaling pathway” (hyperdges $e_1$–$e_7$), “Hypoxic and oxygen homeostasis regulation of HIF-1-$\alpha$” (hyperedges $e_9, e_{10}$), and “Notch-mediated HES/HEY network” (hyperedges $e_8, e_{11}$). Hypoxia-inducible factor 1 (HIF-1) is a heterodimeric transcription factor that regulates genes that are induced by hypoxia [36]. It is a complex of HIF-1$\alpha$ (HIF1A) and HIF-1$\beta$ (aryl hydrocarbon receptor nuclear translocator or ARNT). “Hairy/enhancer-of-split related with YRPW motif protein 2” (HEY2) is a transcriptional repressor [37] that physically interacts with ARNT (hyperedge $e_{11}$). The hyperdges $e_9$ and $e_{11}$ show a pair of reactions where HIF1 is formed and then repressed by HEY2. Hyperedges $e_1$–$e_7$ capture events in the Notch signaling pathway that occur upstream of the formation of the transcriptional activator formed by the complex of the nuclear protein “Recombining binding protein suppressor of hairless” (RBPJ) and Notch intracellular domain (NICD). The expression of protein HEY2 is up-regulated by the NICD/RBPJ complex [38].

This signaling hypergraph was markedly smaller than the other two examples. The hypergraph had 6169 forward-reachable hyperedges, but only 23 hyperedges were backward-traceable, hence only 23 hyperedges were doubly-reachable, due to the poor connectivity of the HEY2/ARNT complex to other vertices in the graph. Even though the hypergraph is small, the hyperpath shown is not the only shortest hyperpath to the target, as $e_2$ and $e_3$ can be replaced by hyperedges containing Jagged2 instead of Jagged1.

## Conclusions

We have presented the first heuristic for Shortest Hyperpaths in general directed hypergraphs with positive edge weights, where the length of a hyperpath is the sum of the weights of its hyperedges. The heuristic handles cycles, is guaranteed to be efficient, finds optimal hyperpaths for singleton-tail hypergraphs, and is highly accurate in practice. It matches the state-of-the-art mixed-integer linear program for shortest acyclic hyperpaths on over  99% of all instances from the NCI-PID and Reactome databases, and surpasses the state-of-the-art on all instances where no acyclic hyperpath exists. Moreover, exhaustively enumerating all source-sink hyperpaths using our hyperpath enumeration algorithm demonstrates that on every cyclic instance from these databases, the heuristic was provably optimal.

### Further research

Given that we can quickly find hyperpaths that are close to optimal in real cell-signaling hypergraphs, several research directions beckon. While the inapproximability of Shortest Hyperpaths [16] rules out a constant-factor approximation unless  $\text {P} \!=\! \text {NP}$, is there an approximation algorithm whose *approximation ratio* on hypergraphs with *n* vertices matches the theoretical lower bound of $\ln n$? More practically, given that in our experiments our heuristic was suboptimal only on acyclic instances, is there a fast method for *acyclic hyperpaths* that outperforms our heuristic? Since a user would like to know how close to optimal a computed hyperpath is for their particular input graph, is there an efficient heuristic that, as well as giving an upper bound on the optimum through its hyperpath, also outputs a *lower bound* on the length of the shortest hyperpath? Many intriguing research avenues are open.

## Data Availability

Source code for the hyperpath heuristic and the hyperpath enumeration algorithm, as well as the hypergraphs from the parsed Reactome, Small, Medium, and Large datasets, is available free for non-commercial use at http://hhugin.cs.arizona.edu.
