# Correction: Heuristic shortest hyperpaths in cell signaling hypergraphs

**DOI:** 10.1186/s13015-022-00222-y

**Published:** 2022-12-29

**Authors:** Spencer Krieger, John Kececioglu

**Affiliations:** Department of Computer Science, The University of Arizona, Tucson, AZ 85721 USA

## Correction: Algorithms for Molecular Biology (2022) 17:12 https://doi.org/10.1186/s13015-022-00217-9

Following the publication of the original article [1], the authors identified the errors on the formatting issues of definitions, lemmas, theorems, and mathematical proofs throughout the paper.

The formatting issues have been corrected in the original version of the article. Please access the link given below to view the updated original article.

There are no scientific content errors noted in the article.
